# The gut microbiome and metabolites are altered and interrelated in patients with functional constipation

**DOI:** 10.3389/fmicb.2023.1320567

**Published:** 2023-12-06

**Authors:** Yan-qiu Li, Xiang-yun Yan, Xian-jun Xiao, Pei-tao Ma, Si-qi Wang, Hui-lin Liu, Wei Zhang, Min Chen, Jun-peng Yao, Ying Li

**Affiliations:** ^1^Acupuncture and Tuina School, Chengdu University of Traditional Chinese Medicine, Chengdu, Sichuan, China; ^2^School of Health Preservation and Rehabilitation, Chengdu University of Traditional Chinese Medicine, Chengdu, Sichuan, China; ^3^Anorectal Department, Hospital of Chengdu University of Traditional Chinese Medicine, Chengdu, Sichuan, China

**Keywords:** functional constipation, gut microbiota, fecal metabolites, correlation analysis, clinical trial

## Abstract

**Introduction:**

Gut microbiota and metabolites have been identified to contribute to the pathogenesis of functional constipation (FC); however, the underlying mechanism(s) have not been elucidated, and the relationship between the gut microbiota and metabolites in FC has received limited attention in the literature.

**Methods:**

16S rDNA sequencing and non-targeted metabolomic detection based on liquid chromatography-mass spectrometry (LC–MS/MS) technologies were combined to analyze the altered gut microbiome and metabolic profile of fecal samples from FC patients and healthy individuals (healthy control; HC).

**Results:**

The richness and diversity of gut microbiota significantly (*p* < 0.01) increased in FC patients. Compared to the HC group, 18 genera, including *Intestinibacter, Klebsiella*, and *Akkermansia*, exhibited statistically significant changes (*p* < 0.05). Metabolic analysis showed that metabolic profiles were also markedly altered with 79 metabolites, such as (-)-caryophyllene oxide, chenodeoxycholic acid, and biliverdin, indicating significant inter-group differences (*p* < 0.05). Besides, the primary bile acid biosynthesis, as well as the metabolic profile of porphyrin and chlorophyll, were the most dominant enriched pathways (FDR < 0.01), in which chenodeoxycholic acid and biliverdin were significantly enriched, respectively. Correlation analysis demonstrated a strong relationship between 10 genera and 19 metabolites (*r* > 0.6, FDR < 0.05), and notably, *Intestinibacter* showed a negative correlation with biliverdin (FDR < 0.001), which highlighted the interplay of the gut microbiota and metabolites in the pathogenesis of FC.

**Conclusion:**

Our research describes the characteristics of the gut microbiota and metabolic profiles and the correlation between the gut microbiota and metabolites in FC patients. This may contribute to the understanding of the underlying mechanisms involved in FC pathogenesis and may provide novel insights into therapeutic interventions.

## Introduction

Functional constipation (FC) is an incurable gastrointestinal disorder characterized by recurrent reduced bowel movements, straining, and hard feces (Lacy et al., 2016). It is estimated that ~11.7% of the global population suffers from FC, and it is more prevalent in women (Barberio et al., 2021). In addition to paying for expensive medical care (Johanson and Kralstein, 2007), patients tend to face lower quality of life (Koloski et al., 2013) and higher risks of severe diseases such as colorectal cancer (Sumida et al., 2019). Moreover, the absence of targeted therapeutic interventions is attributed to the intricate and yet poorly understood pathophysiology of FC. Currently, laxatives are a favorable choice for alleviating relative symptoms of FC (Liu et al., 2016). However, frequent usage of laxatives can result in drug dependence and a cathartic colon; therefore, a laxative is not a satisfactory therapy for FC patients (Yao et al., 2022). Therefore, there is a perceived need to perform further research into the mechanism of FC, with the aim of enhancing the efficacy of treatment strategies.

The gut microbiome, resident in the intestinal tract of humans, establishes a mutualistic and symbiotic relationship with the host and can influence the physiology and pathology of the intestine (Reigstad and Kashyap, 2013). Although the mechanism of FC caused by gut microbiota is not well-known, researchers have reached an agreement that restoring the balance of gut microbiota can be beneficial for patients with FC (Wu et al., 2023). It has been shown that the manipulation of the gut microbiome has the potential to decrease the presence of inflammation factors and alleviate inflammatory reactions (Fontaine et al., 2015; Stein, 2021). Additionally, this manipulation has shown promise in restoring the integrity of the intestinal mucosal barrier and promoting regular movement of the intestinal tract (Fu et al., 2021). Besides, studies have discovered that *Bifidobacterium longum* and *Lacticaseibacillus rhamnosus* could soften stools and relieve the symptoms of FC (Lai et al., 2023). Therefore, FC management can be facilitated by analyzing the features of gut microbiota and elucidating the mechanism of FC mediated by the gut microbiome.

Gut metabolites, produced by the gut microbiota and host, help maintain gut microenvironment stability and physiological function. Correspondingly, the presence of aberrant gut metabolites can lead to impaired functioning of the gut microbiota and contribute to the development of several intestinal disorders. Studies have revealed that short-chain fatty acids (SCFAs), the common metabolites produced by the gut microbiota, can provide energy for intestinal epithelial cells and promote the contraction of intestinal smooth muscle to improve intestinal movement (Wang et al., 2019). Bile acids, crucial metabolites present in the human gut, possess the ability to modulate gut motility and impede the excessive proliferation of the gut microbiome, hence preserving the integrity of the gut mucosal barrier (Hofmann and Eckmann, 2006; Bunnett Nigel, 2014). Researchers have reported that regulating the abundance of these metabolites helps to improve the relative symptoms of FC (Liu et al., 2022; Jeong et al., 2023).

In short, dysregulation of the gut microbiota and associated metabolites constitutes significant elements in the development of FC and needs additional research. However, the available literature has mainly explored the pathogenesis of FC in terms of the gut microbiota, while the interaction of the microbiome and metabolites in the development of FC has been less reported. Therefore, we employed a combination of 16S rDNA sequencing and non-targeted metabolomic detection based on liquid chromatography-mass spectrometry (LC–MS/MS) technologies to analyze the alterations of the gut microbiome and metabolites in FC patients and explore the interactions of the differential gut microbiome and metabolites, with the ultimate goal of providing more support for future research on the mechanism and treatment of FC.

## Methods

### Recruitment and sample collection

Twenty-one FC patients were recruited from January 2022 to October 2022. Patients were diagnosed according to the Rome IV diagnostic criteria and recruited at the outpatient of the Affiliated Hospital of Chengdu University of Traditional Chinese Medicine (Sichuan Province, China). The inclusion criteria of patients were as follows: (1) diagnosed with FC by physicians according to the Rome IV criteria; (2) aged 18 to 60 years old; (3) not taking any drugs for FC for at least 1 week before recruitment; (4) maintaining regular eating habits and no extreme diets like vegetarian food; and (5) not suffering from severe anxiety, depression, or other psychological illness. In addition, 21 healthy individuals were mainly enrolled online (WeChat platform). We used online questionnaires and face-to-face communication to screen healthy individuals with the following requirements: (1) aged 18–60 years old; (2) complete spontaneous bowel movements (CSBMs) once a day and 6–7 times a week; (3) a fecal trait score of 4 according to Bristol scoring criteria; (4) defecation without abdominal pain or other discomforts; (5) not suffering from intestinal disorders or other diseases that can affect the intestinal flora; and (6) no extreme diets like vegetarian food. All FC patients and healthy individuals were to be excluded if they (1) received any probiotics or antibiotics or other therapies that may disturb the gut microbiota within a month before stool sample collection; (2) had any other diseases with gastrointestinal involvement; (3) had serious primary diseases such as progression cancer, severe cardiovascular diseases, or cognitive dysfunction, or were unable to cooperate; and (4) were pregnant or lactating women. Finally, written informed consent from all participants was obtained. This study was approved by Sichuan Provincial Commission of Traditional Chinese Medicine Regional Ethics Review (approval ID: 2021KL-023) and registered on the Chinese Clinical Trial Registry (ChiCTR2100048831). Fresh stool samples of all participants were collected in sterile stool containers and immediately transferred to the −80°C freezer.

### DNA extraction and 16s rDNA sequencing

The sodium dodecyl sulfate (SDS) method was used to extract DNA from the fecal samples, and the quality and concentration of DNA were tested by agarose gel electrophoresis. Subsequently, the DNA was diluted to 1 ng/μl with sterile water. PCR amplification was performed on the 16S V3–V4 region. Specific primers with barcode, Phusion^®^ High Fidelity PCR Master Mix with GC Buffer (New England Biolabs), and high-efficiency, high-fidelity enzymes were selected for PCR to ensure amplification efficiency and accuracy. The PCR products were detected by 2% agarose gel electrophoresis, and equal amounts of samples were mixed according to the PCR product concentration, and then the PCR products were detected again. After that, the strips were recovered with an adhesive recovery kit (Qiagen). The Next^®^ Ultra™ The IIDNA Library Preparation Kit (New England Biolabs) was used for library construction, which was subjected to test the Qubit and Q-PCR quantification. After the library was qualified, NovaSeq6000 was used for machine sequencing.

### 16s rDNA sequencing result analysis

After gaining the effective tags, the DADA2 module in QIIME2 software was used for noise reduction and filtering out sequences to obtain the final ASVs (Amplicon Sequence Variants) and feature table. Subsequently, the classify sklearn module in QIIME2 software was used to compare the obtained ASVs with the database to get species information for each ASV. According to the SILVA (v132) database, effective sequences were clustered into operational taxonomic units (OTUs) with 97% consistency, and the species annotation analysis was performed. The QIIME2 software (Version 1.9.1) was used to calculate the alpha diversity, including the Chao1 index, Shannon index, and Simpson index, and generate the rarefaction curve. An independent sample *t*-test was used to compare the inter-group difference of indices between the two groups, and Graphpad Prism software (Version 8) was used to generate a bar graph. R package (version 4.3.0) was used to perform the principal coordinates analysis (PCoA). The linear discriminant analysis (LDA) effect size (LEfSe) software was used to analyze and screen out the significantly differential microbiome, and the differential microbiome with LDA-value > 3 was selected. Finally, Spearman's correlation analysis was used to explore the correlation between clinical parameters of FC and differential microbiome [significance threshold was FDR (false discovery rate) < 0.05].

### Fecal metabolome profiling

Metabolites were extracted from fecal samples after pretreatment. Fecal metabolites profiling was performed using the untargeted metabolomics approach through LC-MS/MS technology, which combined ultra-performance liquid chromatography (UHPLC) with high-resolution mass spectrometry. The chromatographic column was selected for the chromatographic conditions, the chromatographic column temperature remained at 45°C mobile phase, and the flow rate was kept at 0.2 mL/min. The mass spectrum condition was set as the ESI source, and the signal acquisition was collected in both positive and negative ion scanning modes. The mobile phase consisted of 0.1% formic acid (A) and methyl alcohol (B) in positive ion mode, while the mobile phase was 5 mM ammonium acetate (pH9.0) and methyl alcohol (B) in negative ion mode. The scanning range was set to m/z 100–1,500. The raw data files were imported into the Compound Discoverer 3.1 (Thermo Fisher) software to further perform qualitative and quantitative analysis.

### LC-MS/MS metabolic analysis

Using SIMCA software (version 14.1), the orthogonal partial least-squares discriminant analysis (OPLS-DA) was performed to compare the differences between the two groups. The variable importance in projection (VIP) value of the first principal component, fold change, and *p*-value of *t*-test were calculated to screen out the significantly differential metabolites. Using the R package (version 4.3.0), volcano plots were generated to demonstrate the differential metabolites with fold change > 2, VIP value > 1.5, and *p* < 0.05. Kyoto Encyclopedia of Genes and Genomes (KEGG) pathway enrichment analysis was conducted to evaluate the relationship between the differential metabolites and different metabolic pathways via the MetaboAnalyst platform (https://www.metaboanalyst.ca/MetaboAnalyst/). Spearman's correlation analysis and Mantel test were used to explore the correlation between differential metabolites and clinical parameters of FC (significance threshold was FDR < 0.05).

### Statistical analysis

Statistical analysis was performed using SPSS software (version 25.0), SIMCA software (version 14.1), GraphPad Prism software (version 8), and R package (version 4.3.0). Continuous variables with homogeneity of normal variance were expressed as mean ± SD and an independent sample *t-*test was used to compare the difference between the two groups, but the Wilcoxon rank-sum test was used for normal variables with unequal variances. Generally, *p* < 0.05 was considered statistically significant, and the Benjamini-Hochberg (BH) method was used to calculate the adjusted FDR *p*-value.

Moreover, Spearman's correlation analysis was used to explore the correlation between the differential gut microbiome and differential metabolites (significance threshold was FDR < 0.05). Through Random Forest analysis, the receiver operating characteristic curve (ROC) was drawn, and the area under the curve (AUC) was calculated to evaluate the discriminatory accuracy of the differential genera and differential metabolites.

## Results

### Characteristics of participants

In total, 21 patients with FC and 21 HC were enrolled in this study. The demographic and clinical presentation data are presented in Table 1. The average age and body mass index (*p* > 0.05) were balanced across the two groups, and no significant difference was found. Similarly, they had no significant difference in gender distribution. Compared to the HC group, fewer CSBMs per week, lower levels of bristol stool form scale (BSFS), and higher levels of straining during defecation were identified in the FC group (*p* < 0.01), and all these were the dominant manifestations of FC. Besides, the self-assessment of constipation quality of life (PAC-QOL) index, self-rating anxiety scale (SAS) index, and self-rating depression scale (SDS) index were much higher in the FC group than in the HC group (*p* = 7.02 × 10^−9^, *p* = 3.57 × 10^−7^, *p* = 2.06 × 10^−6^, respectively).

**Table 1:** Demographic and clinical presentation data of participants.

**Variables**		**FC group**	**HC group**	***p-*value**
Age (means ± SD)		32.71 ± 9.62	27.90 ± 7.58	0.08
Gender, n	Female	21	21	-
	Male	0	0	
Body mass index (means ± SD)		20.67 ± 2.57	21.60 ± 2.39	0.27
Disease course (months) (means ± SD)		132.29 ± 73.79	-	-
CSBMs/week (means ± SD)		0.71 ± 0.78	7.48 ± 1.66	8.77 × 10^−18^
BSFS (means ± SD)		2.74 ± 0.84	4.10 ± 0.40	5.09 × 10^−6^
Straining during defecation (means ± SD)		1.04 ± 0.33	0.12 ± 0.21	3.29 × 10^−8^
PAC-QOL index (means ± SD)		2.57 ± 0.61	1.14 ± 0.09	7.02 × 10^−9^
SAS index (means ± SD)		42.26 ± 6.96	27.16 ± 6.58	3.57 × 10^−7^
SDS index (means ± SD)		43.33 ± 8.88	27.89 ± 5.63	2.06 × 10^−6^

### Alterations of the gut microbiome community associated with FC

**Figure 1:**
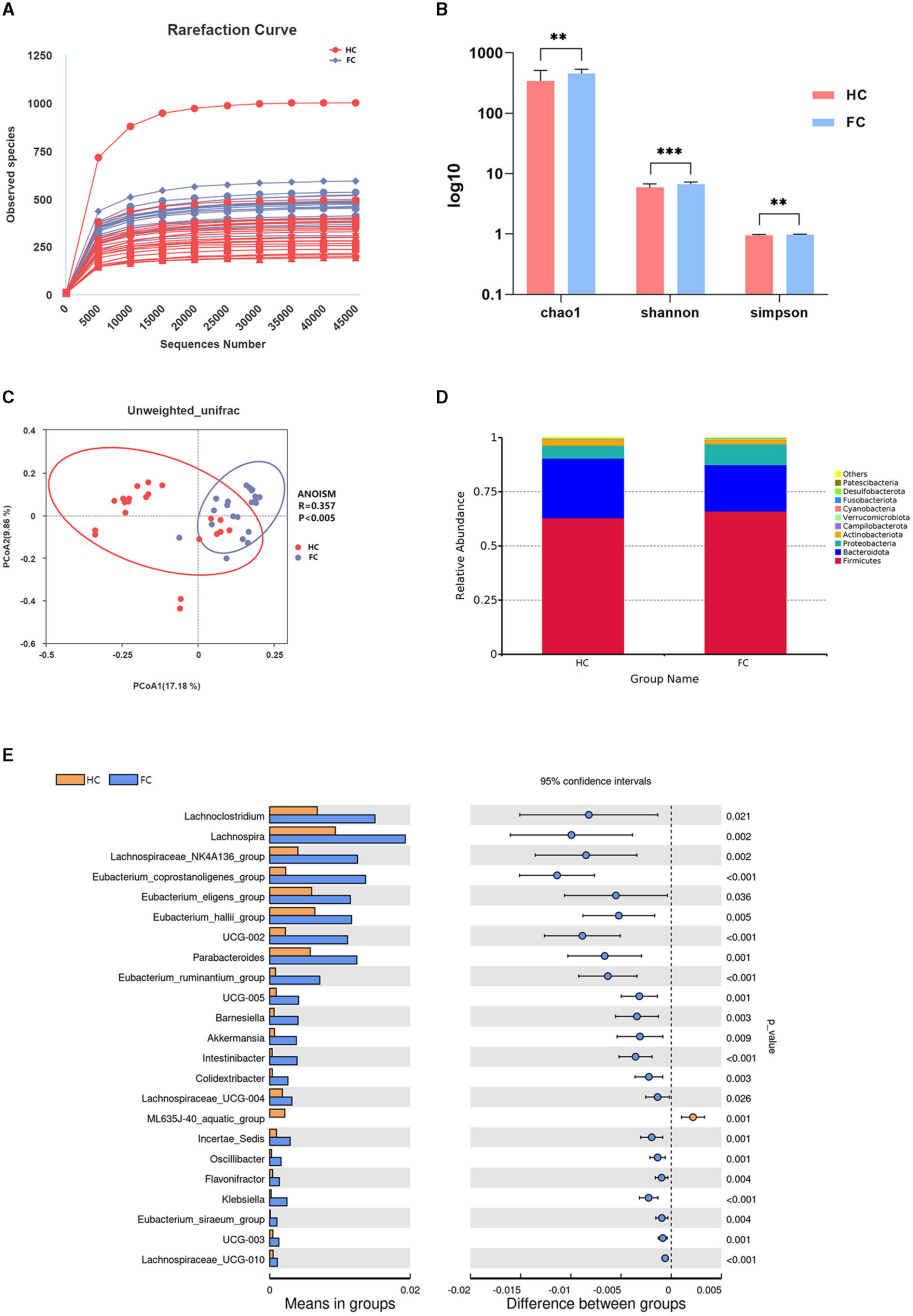
**(A)** Rarefaction curve. Each curve represents a sample. The nearly smooth curves indicate that sequencing samples are adequate. **(B)** Inter-group differences of alpha diversity indices, including the Chao1 index, Shannon index, and Simpson index. ^***^p < 0.001, ^**^p < 0.01. **(C)** PCoA plot showing the difference in the fecal microbial composition between the two groups. The abscissa and ordinate represent the two main components with the greatest contribution to explaining the inter-group difference. **(D)** Relative abundance of the microbiome at the phylum level of the two groups. **(E)** Differential microbiome of the FC and HC groups at the genus levels.

Annotated with the SILVA (v132) database, a total of 5,077 OTUs were obtained with 97% similarity. A rarefaction curve based on observed species suggested the gut microbial richness was high in the FC group (Figure 1A). Additionally, the alpha diversity indices, including the Chao1 index, Shannon index, and Simpson index, also indicated that the microbial diversity in the FC group was significantly high (*p* < 0.01) (Figure 1B). The principal coordinates analysis (PCoA) showed a significant difference in beta diversity between the two groups, and ANOISM analysis revealed that the gut microbial community in the FC group changed appreciably (R = 0.357, *p* < 0.005) (Figure 1C).

The gut microbial structure at the phylum level in these two groups was analyzed, and the top 10 dominant taxa are presented in Figure 1D. Specifically, *Firmicutes* and *Bacteroidota* were the dominant taxa at the phylum level in these two groups, and there was no significant inter-group difference (*p* > 0.05). Among the 10 dominant phyla, the abundance of *Verrucomicrobiota* increased markedly in the FC group (*p* < 0.01). The significant differential genera between these two groups at the genus level are shown in Figure 1E. We noticed that *Eubacterium_coprostanoligenes_group, Oscillospiraceae_UCG-002, Eubacterium_ruminantium_group, Akkermansia, Klebsiella, Parabacteroides*, and *Lachnospiraceae_UCG-010* (*p* < 0.001) were significantly enriched in the FC group, while *ML635J-40_aquatic_group* was significantly enriched in the HC group (*p* = 0.001).

**Figure 2:**
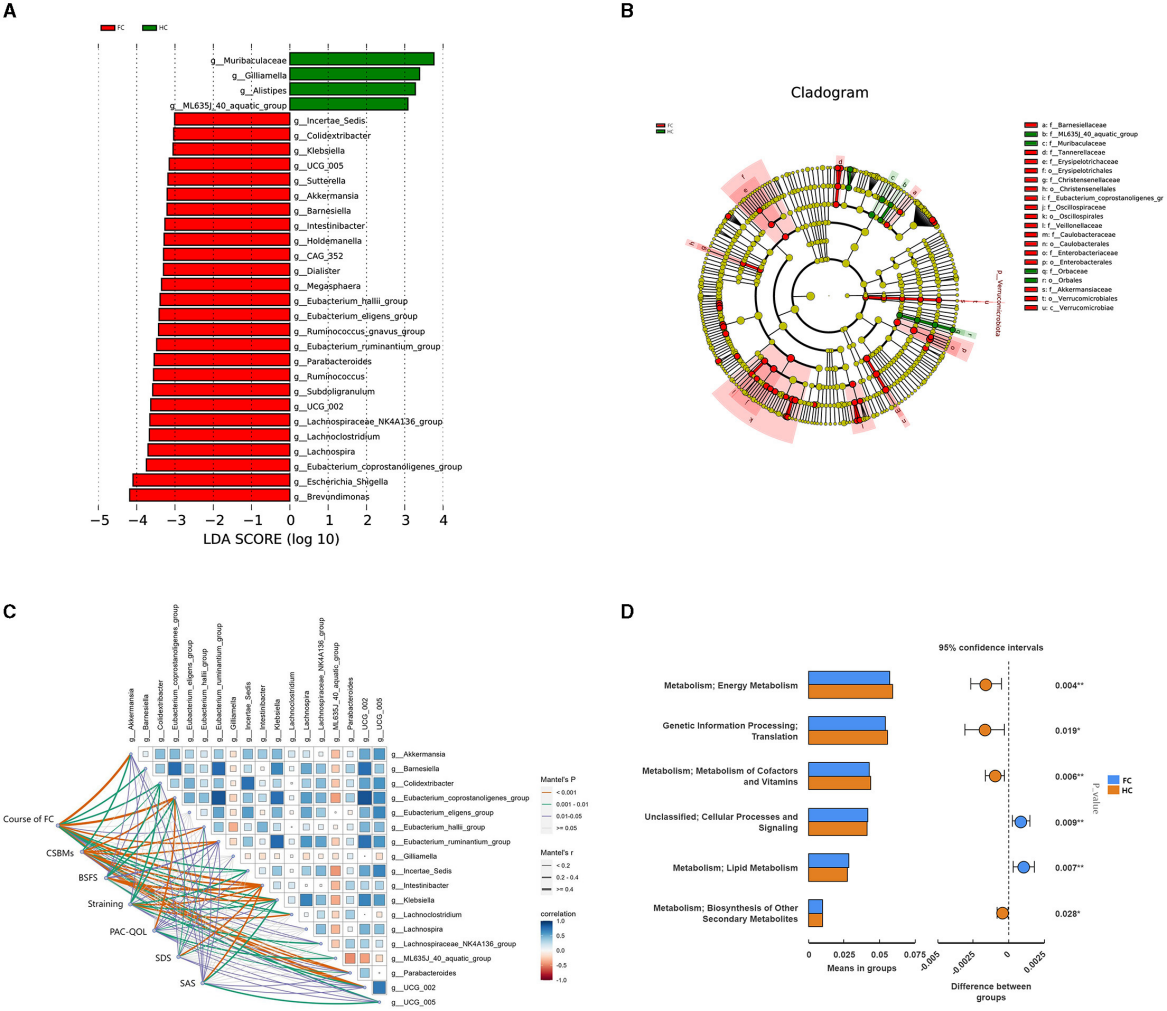
**(A)** LDA score diagram and the significantly differential genera. The color represents groups, and the length of the histogram represents the LDA score. The higher the score, the more significant difference in the differential microbiome. **(B)** Phylogenetic tree in the cladogram of differential taxa. Different colors represent different groups and different microbiota at different levels. **(C)** Pairwise comparisons of the gut genera with Spearman's correlation coefficient and the correlation between genera and clinical parameters with the Mantel test. The edge width represents Spearman's r-value for the corresponding distance correlations, and the color represents the statistical significance. Blue represents a positive correlation, and red represents a negative correlation. **(D)** PICRUST result indicating the potential function of differential microbiota. Red represents the HC group, and blue represents the FC group.

LEfSe analysis was conducted to further identify the key differential genera between the two groups, and 30 differential genera with LDA value > 3 were finally identified (Figures 2A, B). Further, 18 genera were identified to be the significantly differential microbiota through the *t*-test analysis (*p* < 0.05) (Supplementary Table S1). The dominant genera with higher abundance in the FC group were *Eubacterium_coprostanoligenes_group, Intestinibacter, Klebsiella, Eubacterium_ruminantium_group, Colidextribacter*, and *Akkermansia*. While *Gilliamella, ML635J_40_aquatic_group*, and *Parabacteroides* had higher abundance in the HC group.

To further investigate the associations between the gut microbiota and manifestations of FC, mantel tests were performed to demonstrate the correlation between the 18 differential genera and clinical indicators (Figure 2C). The result showed that these 18 genera were all significantly correlated with clinical parameters of FC (FDR < 0.05), and 15 genera were significantly correlated with CSBMs and BSFS, among which *Intestinibacter, Klebsiella, Eubacterium_coporstanoligenes_group*, and *Colidextribacter* enriched in the FC group were negatively correlated with CSBMs, BSFS, and PAC-QOL, and positively correlated with the course of FC, straining, SDS and SAS. *ML635J_40_aquatic_group* and *Gilliamella* showed the opposite correlation tendency (Supplementary Table S2). Besides, we could notice that 13 genera were significantly correlated with all these clinical indicators, suggesting these genera could not only have an impact on gut function but also the emotional state.

### The potential function of the gut microbiome in the progress of FC

Besides, PICRUST was utilized to predict the potential function of the differential gut microbiota in FC. The result indicated that the differential gut microbiota could exert an effect on the energy metabolism, metabolism of cofactors and vitamins, lipid metabolism, and biosynthesis of other secondary metabolites (p < 0.05) (Figure 2D). After *p*-value adjustment, lipid metabolism and metabolism of cofactors and vitamins were still significant (FDR < 0.05) (Supplementary Table S3), which indicated that the change in the gut microbiome might have altered the lipid metabolism and metabolism of cofactors and vitamins in the progress of FC.

### Alterations of fecal metabolites profiling associated with FC

**Figure 3:**
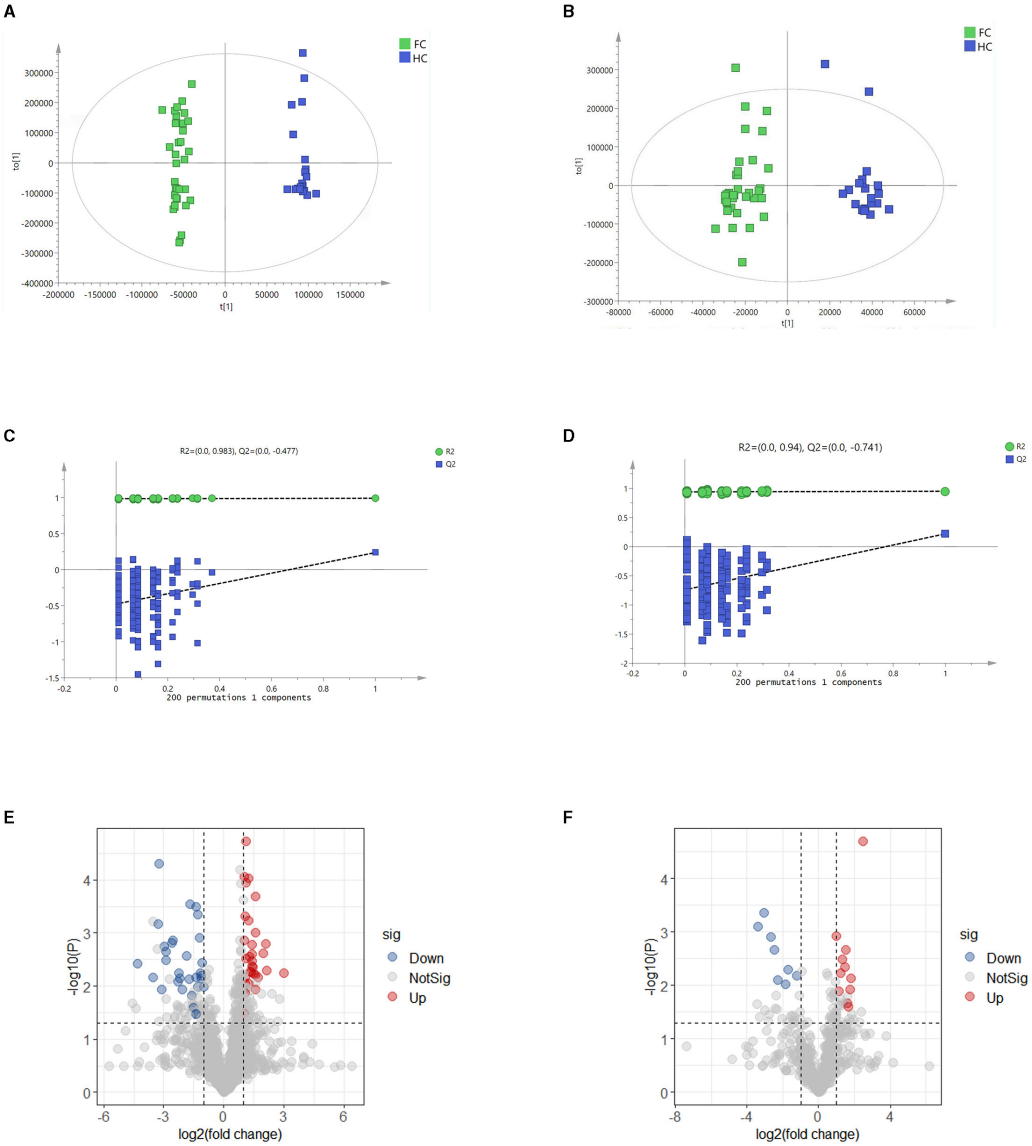
**(A, B)** OPLS-DA score plots in positive and negative ion models, respectively. The abscissa represents the difference between groups, and the ordinate represents the difference within groups. **(C, D)** Validation models through 200 permutation tests in positive and negative ion models, respectively. R2 and Q2 are the intercept values of the fitted lines on the ordinate, respectively, and represent the reliability and overfitting degree of the model. **(E, F)** Volcano plots in positive and negative models, respectively, indicate the discrimination of fecal metabolites between the two groups. The ordinate shows the -log10 (*p*-value), and the abscissa is the log2 (fold change) value. The screening threshold of significantly differential metabolites was fold change > 2, VIP value > 1.5, and *p*-value < 0.05 or fold change < 0.5, VIP value > 1.5, and *p* < 0.05. Every dot represents a kind of metabolite, and the blue ones represent the significantly down-regulated metabolites, while the red dots represent the significantly up-regulated metabolites. The gray dots represent the metabolites that have no significant difference between the two groups.

To identify characteristic metabolites between the two groups, untargeted metabolomics was performed, and forty-two fecal samples (FC, *n* = 21, HC, *n* = 21) were analyzed. OPLS-DA score plots displayed that the fecal metabolites differed significantly between the two groups in both positive and negative ion modes (Figures 3A, B). The 200 permutation test results verified that the OPLS-DA models were not overfitting (Figures 3C, D).

A total of 1,725 metabolites were identified, including 1,202 in positive ion mode and 523 in negative ion mode. Significantly differential metabolites between the two groups were screened by variable importance of projection (VIP) value, fold change, and *p*-value of *t*-test (fold change > 2, VIP value > 1.5, and p < 0.05 or fold change < 0.5, VIP value > 1.5, and p < 0.05). The results are shown in the volcano plots (Figures 3E, F). There were 42 markedly up-regulated metabolites and 39 markedly down-regulated metabolites in total (Supplementary Table S4). (-)-Caryophyllene oxide, 11-deoxy prostaglandin F1α, 3-(4-pyridyl methylidene) chroman-4-one, biliverdin, and chenodeoxycholic acid had higher abundance in the HC group, while glutaric acid, pipecolic acid, N-(2,4-Dimethylphenyl) formamide, and prostaglandin E2 had higher abundance in the FC group.

We then correlated the differential metabolites with clinical indicators to determine the association between differential fecal metabolites and the development of FC. The result showed that there were 78 differential metabolites significantly correlated with clinical parameters of FC (Supplementary Figure S1), and 57 metabolites were identified to be the main correlated differential metabolites with clinical parameters (*r* > 0.5, FDR < 0.05) (Supplementary Table S5). We could notice that N-(2,3-dihydro-1,4-benzodioxin-6-yl)-2,5-dimethyl-3-furamide, norverapamil, pipecolic acid, soyasaponin I, and biliverdin had the most significant correlations with FC. Besides, metabolites abundant in the HC group, such as biliverdin, guggulsterone, and chenodeoxycholic acid were positively correlated with CSBMs and BSFS, while metabolites enriched in the FC group such as 2-(3,5-dimethyl-1H-pyrazol-4-yl)-5-methoxy benzoic acid, prostaglandin E2, norverapamil were negatively associated with CSBMs and BSFS, and positively associated with straining.

### Annotation of differential metabolites through KEGG pathway enrichment analysis

**Figure 4:**
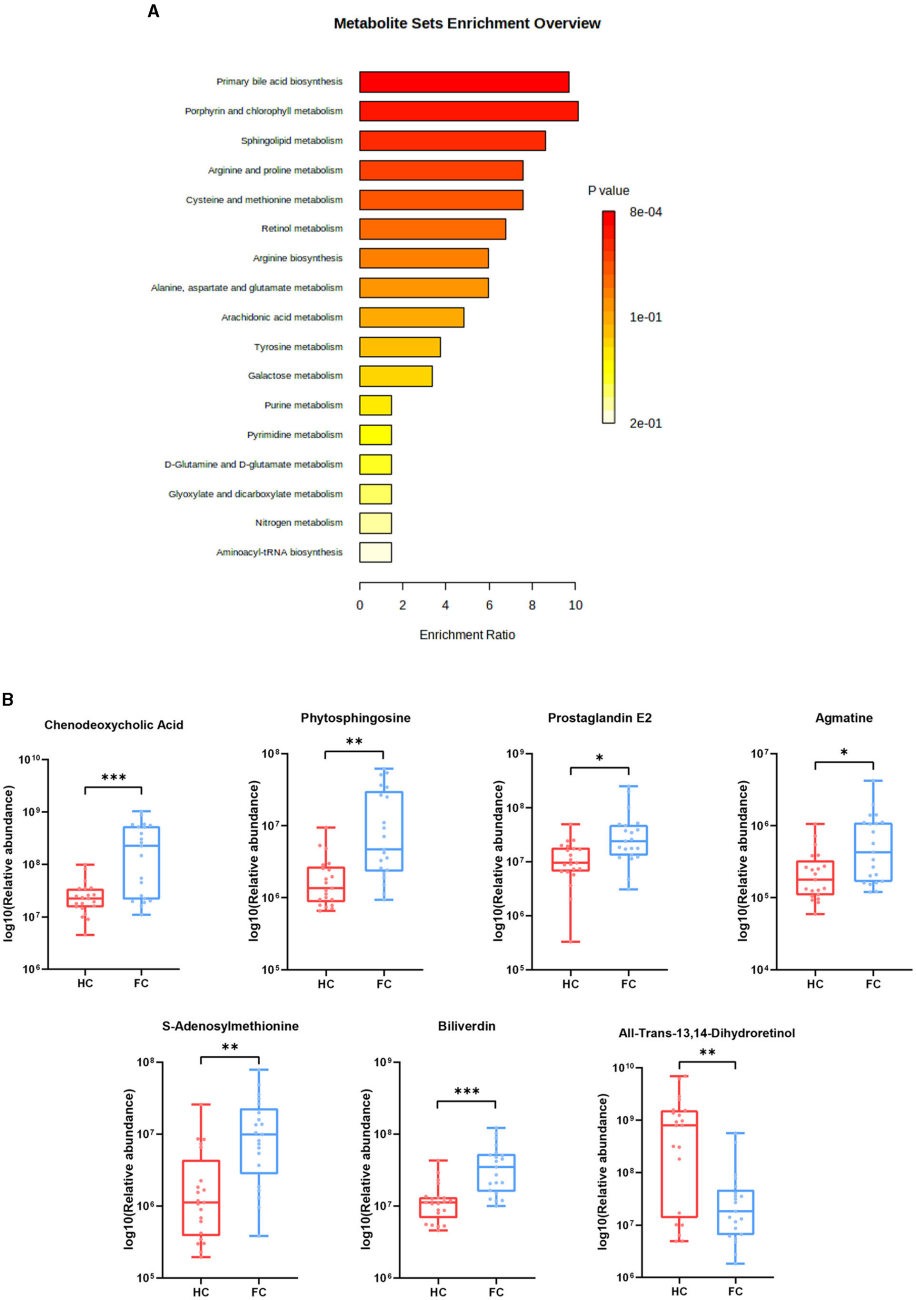
**(A)** Pathway enrichment bar plot of differential metabolites. The abscissa represents enrichment radio, and the ordinate represents enrichment pathways. **(B)** Seven differential metabolites were enriched in the pathways. ***p < 0.001, **p < 0.01, *p < 0.05.

Differential metabolites were further explored through the KEGG pathway enrichment analysis. It was found that differential metabolites were mainly involved in the primary bile acid biosynthesis, sphingolipid metabolism, arachidonic acid metabolism, porphyrin and chlorophyll metabolism, retinol metabolism, arginine and proline metabolism, cysteine and methionine metabolism, arginine biosynthesis alanine, aspartate, and glutamate metabolism (FDR < 0.05) (Figure 4A). Ten metabolites enriched in the above-mentioned pathways and seven significantly altered metabolites are further illustrated in Figure 4B, which exhibited a lower abundance of all-trans-13,14-Dihydroretinol, and a higher abundance of biliverdin, chenodeoxycholic acid, phytosphingosine, S-adenosylmethionine, prostaglandin E2, and agmatine in FC patients.

### Correlation analysis between differential gut microbiota and fecal metabolites

**Figure 5:**
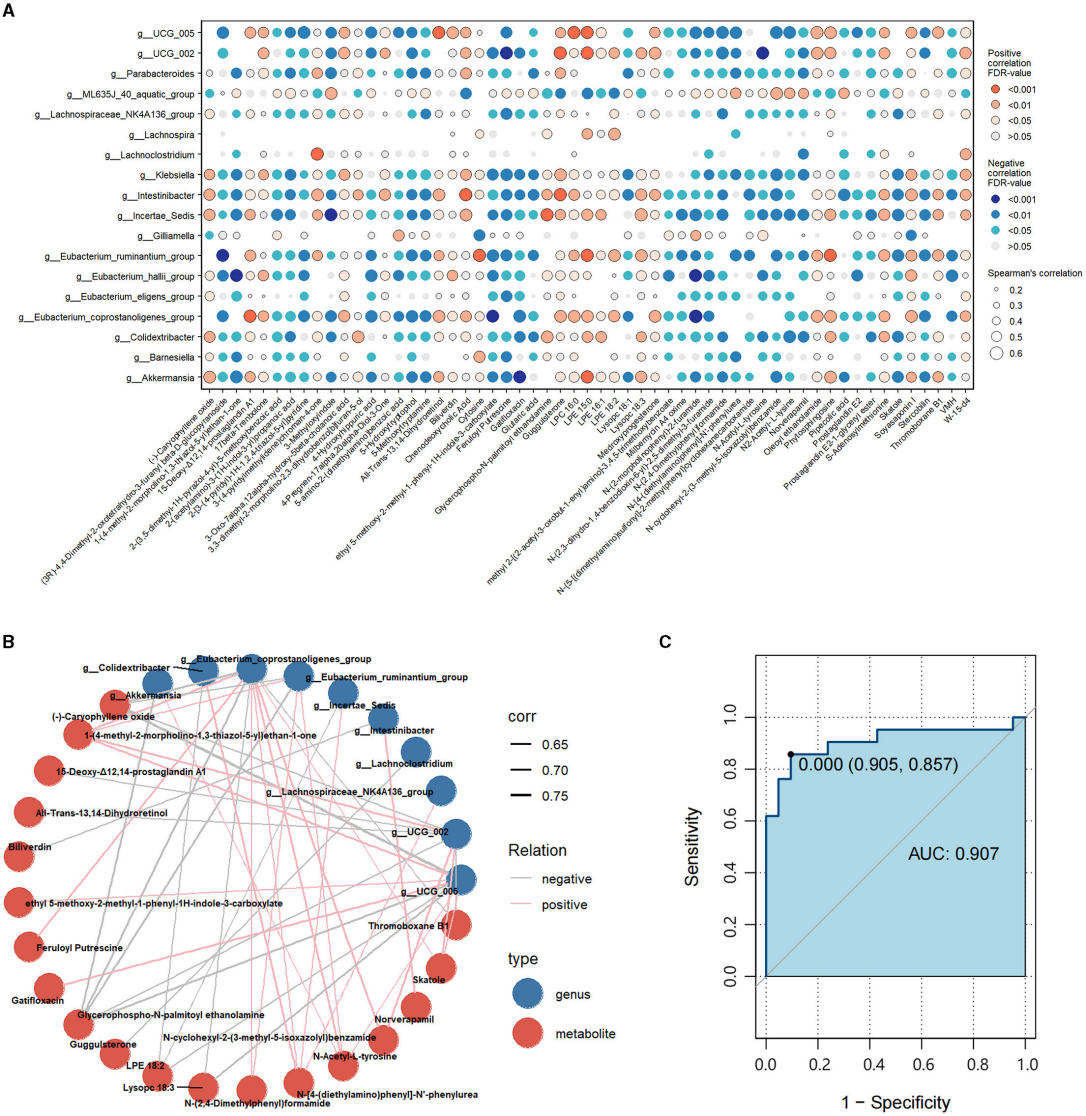
**(A)** Correlations between differential genera and differential metabolites. Red represents a positive correlation, and blue represents a negative correlation. The size of each dot represents the significant degree of correlation. **(B)** Correlation network graph of the differential genera and metabolites with Spearman's *r*-value > 0.6. Blue dots represent the differential genera, and red dots represent differential metabolites. Pink lines represent positive correlations, while gray lines represent negative correlations. The width of the line is proportional to the absolute value of the *r*-value. **(C)** ROC curves of the combination of the differential genera and metabolites with Spearman's *r*-value > 0.6.

To assess the interactions between gut microbiota and metabolites, we correlated the 18 differential genera and 57 differential metabolites (Figure 5A). Then, we constructed a correlation network graph to demonstrate the main interactions of microbiota and microbiome (*r* > 0.6, FDR < 0.05) (Figure 5B). We can observe that there were complex correlations between differential genera and metabolites. *Intestinibacter* was positively correlated with norverapamil (r = 0.665, FDR < 0.001) and negatively correlated with biliverdin (*r* = −0.625, FDR < 0.001). Besides, *Eubacterium_coprostanoligenes_group, Oscillospiraceae_UCG-005, Oscillospiraceae_UCG-002*, and *Eubacterium_ruminantium_group* appeared to be the core differential microbiota. These were found to be negatively correlated with (-)-caryophyllene oxide and glycerophospho-N-palmitoyl ethanolamine, which were the metabolites enriched in the HC group. On the other hand, they were positively correlated with N-Acetyl-L-tyrosine and 1-(4-methyl-2-morpholino-1,3-thiazol-5-yl) ethan-1-one, which were the differential metabolites enriched in the FC group. *Lachnoclostridium* was negatively correlated with guggulsterone (*r* = −0.641, FDR < 0.001) (Supplementary Table S6).

### Function prediction analysis of differential microbiota and metabolites associated with FC

Subsequently, we calculated the AUC values of the differential genera and metabolites. Figure 5C illustrates the differential genera and metabolites with Spearman's r-value > 0.6. The result shows that the combinations of these genera and metabolites had great potential in distinguishing FC patients from HCs with an AUC-value > 0.907 [generally, an AUC value > 0.85 is thought to be a very good value for prediction (Gong et al., 2020)], indicating that the interplay of these genera and metabolites was vital pathogenesis of FC and worthy of further exploration. Besides, we calculated the AUC-values of these 10 genera and 19 metabolites to further identify the key genera or metabolites. It was observed that most differential microbiota had better predictive functions than differential metabolites (Supplementary Tables S7, S8); in particular, *Intestinibacter, Eubacterium_coprostanoligenes_group, Klebsiella, Colidextribacter*, and *Eubacterium_ruminantium_group* with AUC-values ≥0.9 had the highest discrimination ability.

## Discussion

Functional constipation (FC) is a refractory condition that significantly impacts the wellbeing of individuals and diminishes their overall quality of life. Accumulated evidence shows that the alteration of gut microbiota and metabolites is crucial to the pathogenesis of FC. However, the contribution of gut microbiota and associated metabolites to the pathogenesis of FC has not been elucidated. In this study, we recruited 21 patients with FC and 21 healthy individuals to examine the alterations in the gut microbiome and fecal metabolites among FC patients and evaluate the association between the interaction of distinct gut microbiota and fecal metabolites with the progression of FC.

We found that the richness and diversity of gut microbiome significantly differed between patients with FC and healthy individuals, suggesting that gut microbiome dysbiosis occurred in the development of FC. *Intestinibacter, Eubacterium_coprostanoligenes_group, Klebsiella, Akkermansia, Eubacterium_ruminantium_group, Oscillospiraceae_UCG-005*, and *Oscillospiraceae_UCG-002* altered significantly and might be the core genera in the pathogenesis of FC.

*Intestinibacter, Klebsiella*, and *Akkermansia*, recognized as potential pathogens, were shown to be enriched in FC patients, which was consistent with previous research (Cao et al., 2017; Zeyue et al., 2022; Chen et al., 2023). *Intestinibacter* (Forbes et al., 2018; Xiang et al., 2023) and *Klebsiella* (Gevers et al., 2014; Li et al., 2022) were reported to have a close relationship with inflammatory responses in intestinal disorders; therefore, we speculated that the increased abundance of *Intestinibacter* and *Klebsiella* could disrupt the gut function by exerting intestinal inflammation, thus resulting in FC. *Akkermansia* can excessively degrade the mucin, causing dysfunction of the intestinal barrier and accumulation of harmful substances, ultimately leading to the development of intestinal disorders (Zhang et al., 2016). The *Eubacterium_coprostanoligenes_group, Eubacterium_ruminantium_group, Oscillospiraceae_UCG-002*, and *Oscillospiraceae_UCG-005* possess the ability to metabolize dietary fiber through fermentation processes and produce SCFAs which have been associated with various positive effects on gastrointestinal (Mukherjee et al., 2020; Hu et al., 2023) and mental health (Liang et al., 2022; Zhao et al., 2022). However, these genera had a higher abundance in the FC group. Furthermore, we found significant negative correlations between these genera and CSBMs, BSFS, and PAC-QOL and positive correlations between these genera and straining, SAS, and SDS, which suggest that these genera have a detrimental impact on the development of FC.

The aforementioned genera underwent substantial modifications and played a crucial role in diagnostics. They may serve as a key avenue for investigating the mechanism of FC and hold promise as potential therapy targets for FC in the future. In particular, *Intestinibacter* had the best diagnostic efficacy for FC, with the highest AUC = 0.975, and is expected to be a clinical diagnostic biomarker for FC. The findings from PICRUST and KEGG pathway enrichment analysis suggested that the alteration in the gut microbiome could influence the metabolism of lipids, cofactors, and vitamins, which leads us to hypothesize that such effects may arise from the interplay between differential metabolites and the gut microbiome.

In the analysis of metabolites, we discovered the alteration in fecal metabolites in FC patients. Primary bile acid production and porphyrin and chlorophyll metabolism were two of the most important metabolic processes where the differentiated metabolites were engaged. Among the metabolites implicated in these two pathways, chenodeoxycholic acid and biliverdin exhibited differential metabolic pathways. Chenodeoxycholic Acid can modulate the gastrointestinal secretory process and maintain normal defecation (Bazzoli et al., 1983). Consistent with previous research (Wong Banny et al., 2011), chenodeoxycholic acid was found to have a higher abundance in FC patients and was positively correlated with CSBMs, BSFS, and PAC-QOL, while being negatively correlated with straining, SAS, SDS, and the course of FC. Biliverdin was enriched in the HC group and showed a negative correlation with the pathogenesis of FC in our research. Researchers have reported that biliverdin could reduce pro-inflammatory mediators and relieve inflammatory responses (Wegiel et al., 2009; Bonelli et al., 2012). Besides, biliverdin can be reduced into bilirubin, which can alleviate immune inflammatory responses (Vitek et al., 2023) and is thought to be an effective prognostic biomarker for advanced colorectal cancer (Khoei et al., 2020).

Additionally, five notable differential metabolites were involved in other significant pathways. Phytosphingosine was enriched in the HC group and demonstrated a negative correlation with the development of FC. Studies indicated the strong anti-inflammatory activity of phytosphingosine *in vitro* and discovered that it could suppress the inflammatory responses in colitis mice (Montenegro-Burke et al., 2021). Similar to phytosphingosine, agmatine can also inhibit inflammatory responses. Moreover, agmatine has the potential to improve mood disorders (Freitas et al., 2016). In our study, agmatine was enriched in the HC group and was negatively associated with SDS and SAS. Thus, we assumed that agmatine may provide an explanation for the co-occurrence of anxiety or depression in individuals with FC. S-Adenosylmethionine (SAM), a type of bioactive substance involved in various biochemical reactions in the body (Pascale Rosa et al., 2022), had a higher abundance in the HC group. In this study, a positive correlation was observed between SAM and FC pathogenesis. It is possible that the gut microbiome has a self-balancing mechanism at play; however, further studies are needed to verify this assumption. Prostaglandin E2, involved in the arachidonic acid metabolism, can not only regulate the gut inflammatory response and promote the repair of intestinal mucosa but also regulate the expression of aquaporin 3 to regulate intestinal fluid metabolism (Na et al., 2021). In our study, increased levels of prostaglandin E2 were found among FC patients, and we thought that it may be attributed to a mechanism of self-regulation throughout the progression of FC.

In light of the intricate biological dynamics within the human gut, we conducted an analysis to establish the connections between the varied composition of microbiota and metabolites. In the correlation network, *Intestinibacter* was strongly negatively correlated with biliverdin. *Eubacterium_coprostanoligenes_group*, O*scillospiraceae-UCG_005*, O*scillospiraceae-UCG_002*, and *Eubacterium_ruminantium_group* were all negatively correlated with (-)-caryophyllene oxide and glycerophospho-N-palmitoyl ethanolamine, and positively correlated with 1-(4-methyl-2-morpholino-1,3-thiazol-5-yl) ethan-1-one. The abundance of (-)-caryophyllene oxide and glycerophospho-N-palmitoyl ethanolamine, which have been identified as metabolites with potential benefits in anti-inflammatory responses, was decreased in FC patients (Park et al., 2011; Li et al., 2021; Su et al., 2022). Conversely, the concentration of 1-(4-methyl-2-morpholino-1,3-thiazol-5-yl) ethan-1-one was observed to be elevated in the FC group. Despite the limited research on 1-(4-methyl-2-morpholino-1,3-thiazol-5-yl) ethan-1-one, our findings indicate its significant negative association with CSBMs and PAC-QOL. This suggests a potential interaction between 1-(4-methyl-2-morpholino-1,3-thiazol-5-yl) ethan-1-one and the gut microbiome, which may influence both the physiological functioning of the intestinal tract and the psychological wellbeing of patients.

In summary, our study found a complex and close relationship between significantly differential genera and metabolites. These altered genera and metabolites were reported to be related to inflammatory reactions; therefore, we speculated that these genera and their related metabolic profiles might mediate the occurrence of intestinal inflammation, affecting the integrity of intestinal mucosa and intestinal contractile function, thus resulting in FC. Biliverdin can reduce the release of pro-inflammatory factors in the gut of septic rats and improve intestinal contractility (Nakao et al., 2004). Besides, research has reported that biliverdin can be used in inflammatory bowel disease (Berberat et al., 2005). Therefore, biliverdin has the potential to be another therapy for FC by regulating intestinal microbiota and improving the relief of inflammatory reactions in the gut. However, this hypothesis still needs to be verified in future large-sample studies. Additionally, (-)-caryophyllene oxide, glycerophospho-N-palmitoyl ethanolamine, N-[4-(diethylamino)phenyl]-N'-phenylurea, and 1-(4-methyl-2-morpholino-1,3-thiazol-5-yl)ethan-1-one are the most correlated metabolites in the correlation network and might exert a more comprehensive and extensive regulation on the disordered gut microbiota, which are also worth further exploration.

Despite the rigorous adherence to scheduling and implementation in this clinical trial, there were several limitations in this study. Due to the small sample size, the results may be limited for generalization; thus, trials with larger sample sizes are required to verify the suggested associations. Besides, it is well-known that the composition of gut microbiota varies across different age groups. However, the sample size of our research was not sufficient to conduct in-depth research on the differences in the gut microbiota related to aging. Therefore, exploring the changes in the gut microbiota of FC patients at different ages is one of the feasible research directions that may improve clinical treatment. Moreover, it is worth mentioning that we did not confirm the causal relationship between the gut microbiota, metabolites, and clinical parameters in this research, and more studies are needed to further explore this in the future.

## Conclusion

In conclusion, our research demonstrates the characteristics of the gut microbiota and metabolic profiles in FC patients. Investigating the underlying mechanism of FC can yield valuable insights for the improvement of FC treatment strategies. Future research should aim to validate the interaction between the gut microbiota and metabolites in the development of FC. These endeavors may facilitate the discovery of novel therapeutic interventions for individuals affected by FC.

## Data availability statement

The data presented in the study are deposited in the National Library of Medicine (https://submit.ncbi.nlm.nih.gov/) repository, accession number: PRJNA1031121.

## Ethics statement

The studies involving humans were approved by Sichuan Provincial Commission of Traditional Chinese Medicine Regional Ethics Review (Approval ID: 2021KL-023). The participants provided their written informed consent to participate in this study.

## Author contributions

Y-qL: Formal analysis, Methodology, Software, Writing – original draft. X-yY: Data curation, Formal analysis, Software, Writing – review & editing. X-jX: Data curation, Formal analysis, Methodology, Writing – review & editing. P-tM: Methodology, Resources, Writing – review & editing. S-qW: Methodology, Resources, Writing – review & editing. H-lL: Methodology, Resources, Writing – review & editing. WZ: Resources, Validation, Writing – review & editing. MC: Resources, Validation, Writing – review & editing. J-pY: Conceptualization, Project administration, Writing – review & editing. YL: Conceptualization, Funding acquisition, Project administration, Writing – review & editing.
