# Supplementary material for: The gut microbiome and metabolites are altered and interrelated in patients with functional constipation
Source: Front Microbiol. 2023 Dec 6;14:1320567. doi: 10.3389/fmicb.2023.1320567 (PMC10731029; doi:10.3389/fmicb.2023.1320567)
Supplement: Supplementary file 1 [file Data_Sheet_1.docx]

**Supplementary Materials**

**Supplementary Figure**

| **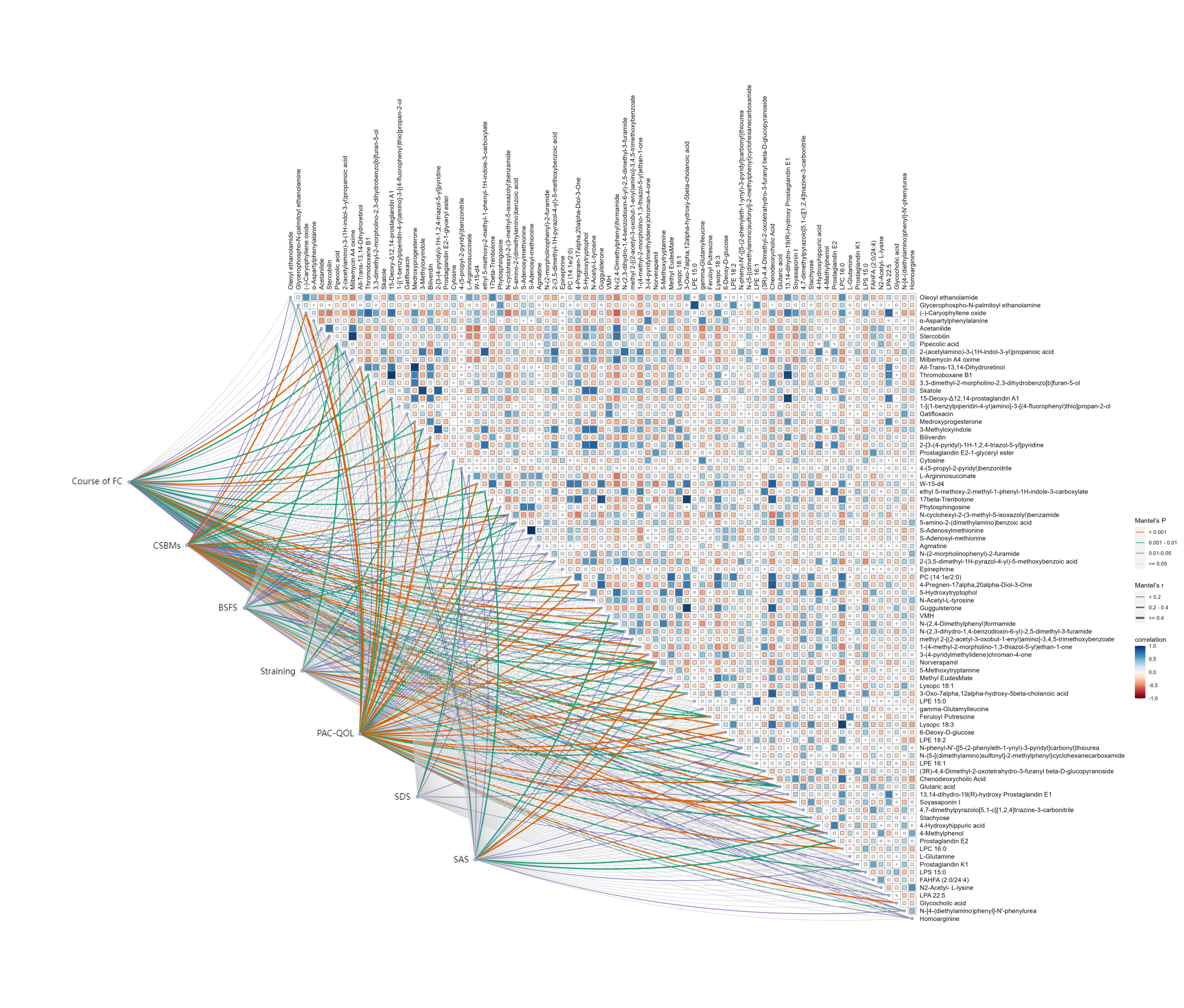** |
| --- |
| **Figure S1** shows the pairwise comparisons of the differential metabolites with Spearman’s correlation coefficient and the correlation between metabolites and clinical parameters with Mantel test. The edge width represents the Spearman’s r-value for the corresponding distance correlations and the color represents the statistical significance. Blue represents a positive correlation and red represents a negative correlation. |

**Supplementary Tables**

| **Table S1**. ***t*-test analysis of the differential gut microbiota with LDA > 3.** | | | |
| --- | --- | --- | --- |
| **Genus** | **Mean-FC** | **Mean-HC** | ***p*-value** |
| *g__Akkermansia* | 0.004 | 0.001 | 0.0091 |
| *g__Alistipes* | 0.008 | 0.009 | 0.7616 |
| *g__Barnesiella* | 0.004 | 0.001 | 0.0033 |
| *g__Brevundimonas* | 0.037 | 0.011 | 0.1463 |
| *g__CAG352* | 0.007 | 0.006 | 0.7950 |
| *g__Colidextribacter* | 0.003 | 0.000 | 0.0031 |
| *g__Dialister* | 0.009 | 0.005 | 0.1880 |
| *g__Escherichia-Shigella* | 0.038 | 0.019 | 0.1017 |
| *g__Eubacterium-coprostanoligenes-group* | 0.014 | 0.002 | 1.52E-06 |
| *g__Eubacteriume-ligens-group* | 0.012 | 0.006 | 0.0355 |
| *g__Eubacterium-hallii-group* | 0.012 | 0.006 | 0.0056 |
| *g__Eubacterium-ruminantium-group* | 0.007 | 0.001 | 0.0002 |
| *g__Gilliamella* | 0.000 | 0.005 | 0.0771 |
| *g__Holdemanella* | 0.005 | 0.001 | 0.3093 |
| *g__IncertaeSedis* | 0.003 | 0.001 | 0.0010 |
| *g__Intestinibacter* | 0.004 | 0.000 | 0.0002 |
| *g__Klebsiella* | 0.002 | 0.000 | 4.42E-05 |
| *g__Lachnoclostridium* | 0.015 | 0.007 | 0.0202 |
| *g__Lachnospira* | 0.019 | 0.009 | 0.0020 |
| *g__Lachnospiraceae-NK4A136-group* | 0.013 | 0.004 | 0.0019 |
| *g__Megasphaera* | 0.005 | 0.000 | 0.2557 |
| *g__ML635J40aquaticgroup* | 0.000 | 0.002 | 0.0008 |
| *g__Muribaculaceae* | 0.012 | 0.019 | 0.6304 |
| *g__Parabacteroides* | 0.012 | 0.006 | 0.0008 |
| *g__Ruminococcus* | 0.018 | 0.010 | 0.0764 |
| *g__Ruminococcusgnavusgroup* | 0.007 | 0.002 | 0.1371 |
| *g__Subdoligranulum* | 0.027 | 0.021 | 0.4023 |
| *g__Sutterella* | 0.009 | 0.005 | 0.1511 |
| *g__UCG002* | 0.011 | 0.002 | 0.0001 |
| *g__UCG005* | 0.004 | 0.001 | 0.0012 |

| **Table S2**. **The correlations between differential genera and clinical parameters.** | | | | |  |
| --- | --- | --- | --- | --- | --- |
| **Genus** | **clinical parameters** | **r-value** | **FDR-value** | **Regulation** | |
| *g__Intestinibacter* | CSBMs | -0.8375 | 5.90E-10 | Negative | |
| *g__Eubacterium_coprostanoligenes_group* | CSBMs | -0.7072 | 3.55E-06 | Negative | |
| *g__Klebsiella* | CSBMs | -0.7017 | 4.05E-06 | Negative | |
| *g__Intestinibacter* | BSFS | -0.6914 | 6.29E-06 | Negative | |
| *g__UCG_002* | CSBMs | -0.6819 | 8.33E-06 | Negative | |
| *g__Eubacterium_ruminantium_group* | CSBMs | -0.6773 | 9.60E-06 | Negative | |
| *g__Colidextribacter* | CSBMs | -0.6748 | 9.64E-06 | Negative | |
| *g__Intestinibacter* | PAC-QOL | -0.6744 | 1.98E-05 | Negative | |
| *g__Eubacterium_coprostanoligenes_group* | PAC-QOL | -0.6598 | 3.14E-05 | Negative | |
| *g__Incertae_Sedis* | CSBMs | -0.6489 | 2.34E-05 | Negative | |
| *g__Klebsiella* | PAC-QOL | -0.6212 | 1.10E-04 | Negative | |
| *g__UCG_005* | CSBMs | -0.6109 | 9.11E-05 | Negative | |
| *g__Akkermansia* | CSBMs | -0.6085 | 9.62E-05 | Negative | |
| *g__ML635J_40_aquatic_group* | Course of disease | -0.6068 | 9.91E-05 | Negative | |
| *g__Klebsiella* | BSFS | -0.5817 | 2.04E-04 | Negative | |
| *g__UCG_005* | PAC-QOL | -0.5815 | 3.62E-04 | Negative | |
| *g__UCG_002* | PAC-QOL | -0.5729 | 4.15E-04 | Negative | |
| *g__Eubacterium_ruminantium_group* | PAC-QOL | -0.5638 | 5.33E-04 | Negative | |
| *g__Lachnospiraceae_NK4A136_group* | CSBMs | -0.5595 | 3.97E-04 | Negative | |
| *g__Parabacteroides* | CSBMs | -0.5583 | 3.97E-04 | Negative | |
| *g__Lachnoclostridium* | BSFS | -0.5434 | 5.60E-04 | Negative | |
| *g__Colidextribacter* | PAC-QOL | -0.5405 | 9.45E-04 | Negative | |
| *g__Parabacteroides* | PAC-QOL | -0.5353 | 9.81E-04 | Negative | |
| *g__Eubacterium_coprostanoligenes_group* | BSFS | -0.5349 | 6.90E-04 | Negative | |
| *g__Lachnoclostridium* | PAC-QOL | -0.5316 | 1.04E-03 | Negative | |
| *g__Parabacteroides* | BSFS | -0.5214 | 9.68E-04 | Negative | |
| *g__Colidextribacter* | BSFS | -0.5182 | 9.81E-04 | Negative | |
| *g__Lachnospiraceae_NK4A136_group* | PAC-QOL | -0.4935 | 2.57E-03 | Negative | |
| *g__Eubacterium_ruminantium_group* | BSFS | -0.4904 | 1.92E-03 | Negative | |
| *g__Incertae_Sedis* | PAC-QOL | -0.4842 | 3.13E-03 | Negative | |
| *g__Barnesiella* | PAC-QOL | -0.4818 | 3.23E-03 | Negative | |
| *g__ML635J_40_aquatic_group* | Straining | -0.4776 | 2.56E-03 | Negative | |
| *g__Akkermansia* | PAC-QOL | -0.4691 | 4.12E-03 | Negative | |
| *g__Akkermansia* | BSFS | -0.4680 | 3.13E-03 | Negative | |
| *g__UCG_002* | BSFS | -0.4566 | 3.93E-03 | Negative | |
| *g__ML635J_40_aquatic_group* | SAS | -0.4492 | 6.04E-03 | Negative | |
| *g__Eubacterium_hallii_group* | CSBMs | -0.4491 | 4.38E-03 | Negative | |
| *g__Incertae_Sedis* | BSFS | -0.4411 | 5.18E-03 | Negative | |
| *g__Eubacterium_hallii_group* | PAC-QOL | -0.4402 | 6.98E-03 | Negative | |
| *g__Lachnoclostridium* | CSBMs | -0.4386 | 5.43E-03 | Negative | |
| *g__ML635J_40_aquatic_group* | SDS | -0.4367 | 7.38E-03 | Negative | |
| *g__Eubacterium_eligens_group* | PAC-QOL | -0.4281 | 8.78E-03 | Negative | |
| *g__Lachnospira* | CSBMs | -0.4266 | 6.79E-03 | Negative | |
| *g__Gilliamella* | Straining | -0.4222 | 7.33E-03 | Negative | |
| *g__Eubacterium_hallii_group* | BSFS | -0.3891 | 1.41E-02 | Negative | |
| *g__Lachnospiraceae_NK4A136_group* | BSFS | -0.3762 | 1.81E-02 | Negative | |
| *g__Eubacterium_eligens_group* | CSBMs | -0.3757 | 1.81E-02 | Negative | |
| *g__Barnesiella* | CSBMs | -0.3709 | 1.97E-02 | Negative | |
| *g__Gilliamella* | SDS | -0.3487 | 3.42E-02 | Negative | |
| *g__UCG_005* | BSFS | -0.3470 | 2.94E-02 | Negative | |
| *g__Gilliamella* | Course of disease | -0.3399 | 3.23E-02 | Negative | |
| *g__Lachnospira* | BSFS | -0.3332 | 3.49E-02 | Negative | |
| *g__Lachnospira* | PAC-QOL | -0.3222 | 4.98E-02 | Negative | |
| *g__Gilliamella* | SAS | -0.3144 | 5.52E-02 | Negative | |
| *g__Barnesiella* | BSFS | -0.2771 | 7.88E-02 | Negative | |
| *g__Eubacterium_eligens_group* | BSFS | -0.1675 | 2.89E-01 | Negative | |
| *g__Gilliamella* | CSBMs | 0.2434 | 1.23E-01 | Positive | |
| *g__Barnesiella* | SDS | 0.2442 | 1.35E-01 | Positive | |
| *g__Barnesiella* | SAS | 0.2511 | 1.25E-01 | Positive | |
| *g__Lachnospiraceae_NK4A136_group* | SDS | 0.2578 | 1.17E-01 | Positive | |
| *g__Barnesiella* | Straining | 0.2804 | 7.57E-02 | Positive | |
| *g__Eubacterium_eligens_group* | Straining | 0.2828 | 7.37E-02 | Positive | |
| *g__Lachnospira* | Straining | 0.2954 | 6.14E-02 | Positive | |
| *g__Eubacterium_eligens_group* | SDS | 0.3149 | 5.52E-02 | Positive | |
| *g__Eubacterium_eligens_group* | SAS | 0.3370 | 3.97E-02 | Positive | |
| *g__Gilliamella* | BSFS | 0.3401 | 3.23E-02 | Positive | |
| *g__Lachnospira* | SAS | 0.3403 | 3.79E-02 | Positive | |
| *g__Lachnoclostridium* | SAS | 0.3460 | 3.49E-02 | Positive | |
| *g__Lachnospiraceae_NK4A136_group* | SAS | 0.3480 | 3.43E-02 | Positive | |
| *g__Parabacteroides* | SAS | 0.3558 | 3.12E-02 | Positive | |
| *g__Eubacterium_eligens_group* | Course of disease | 0.3595 | 2.39E-02 | Positive | |
| *g__Lachnospira* | SDS | 0.3598 | 2.94E-02 | Positive | |
| *g__Gilliamella* | PAC-QOL | 0.3684 | 2.57E-02 | Positive | |
| *g__Parabacteroides* | SDS | 0.3789 | 2.17E-02 | Positive | |
| *g__UCG_005* | Straining | 0.3961 | 1.24E-02 | Positive | |
| *g__Incertae_Sedis* | SDS | 0.4193 | 1.05E-02 | Positive | |
| *g__Lachnospiraceae_NK4A136_group* | Course of disease | 0.4279 | 6.66E-03 | Positive | |
| *g__Lachnospira* | Course of disease | 0.4305 | 6.35E-03 | Positive | |
| *g__Eubacterium_hallii_group* | Straining | 0.4319 | 6.21E-03 | Positive | |
| *g__Lachnospiraceae_NK4A136_group* | Straining | 0.4477 | 4.48E-03 | Positive | |
| *g__Barnesiella* | Course of disease | 0.4507 | 4.27E-03 | Positive | |
| *g__UCG_002* | Straining | 0.4527 | 4.12E-03 | Positive | |
| *g__Parabacteroides* | Straining | 0.4560 | 3.93E-03 | Positive | |
| *g__Lachnoclostridium* | SDS | 0.4696 | 4.12E-03 | Positive | |
| *g__Incertae_Sedis* | SAS | 0.4756 | 3.71E-03 | Positive | |
| *g__Eubacterium_hallii_group* | Course of disease | 0.4759 | 2.60E-03 | Positive | |
| *g__Eubacterium_ruminantium_group* | Straining | 0.4779 | 2.56E-03 | Positive | |
| *g__ML635J_40_aquatic_group* | BSFS | 0.4785 | 2.56E-03 | Positive | |
| *g__ML635J_40_aquatic_group* | PAC-QOL | 0.4837 | 3.13E-03 | Positive | |
| *g__Akkermansia* | Straining | 0.4964 | 1.66E-03 | Positive | |
| *g__UCG_002* | SAS | 0.5018 | 2.20E-03 | Positive | |
| *g__Lachnoclostridium* | Straining | 0.5129 | 1.06E-03 | Positive | |
| *g__Incertae_Sedis* | Straining | 0.5145 | 1.04E-03 | Positive | |
| *g__UCG_002* | SDS | 0.5269 | 1.16E-03 | Positive | |
| *g__Eubacterium_hallii_group* | SAS | 0.5349 | 9.81E-04 | Positive | |
| *g__Eubacterium_hallii_group* | SDS | 0.5360 | 9.81E-04 | Positive | |
| *g__Colidextribacter* | SDS | 0.5362 | 9.81E-04 | Positive | |
| *g__Eubacterium_ruminantium_group* | SDS | 0.5380 | 9.80E-04 | Positive | |
| *g__Klebsiella* | SAS | 0.5456 | 8.27E-04 | Positive | |
| *g__Klebsiella* | SDS | 0.5486 | 7.70E-04 | Positive | |
| *g__Lachnoclostridium* | Course of disease | 0.5569 | 3.97E-04 | Positive | |
| *g__Eubacterium_coprostanoligenes_group* | SAS | 0.5572 | 6.15E-04 | Positive | |
| *g__Eubacterium_coprostanoligenes_group* | SDS | 0.5580 | 6.11E-04 | Positive | |
| *g__Eubacterium_coprostanoligenes_group* | Straining | 0.5580 | 3.97E-04 | Positive | |
| *g__Colidextribacter* | SAS | 0.5705 | 4.37E-04 | Positive | |
| *g__UCG_005* | SDS | 0.5749 | 3.97E-04 | Positive | |
| *g__Incertae_Sedis* | Course of disease | 0.5822 | 2.04E-04 | Positive | |
| *g__Colidextribacter* | Straining | 0.5889 | 1.72E-04 | Positive | |
| *g__UCG_005* | Course of disease | 0.5906 | 1.66E-04 | Positive | |
| *g__Eubacterium_ruminantium_group* | SAS | 0.5912 | 2.67E-04 | Positive | |
| *g__Akkermansia* | SAS | 0.5922 | 2.65E-04 | Positive | |
| *g__Akkermansia* | SDS | 0.6027 | 1.99E-04 | Positive | |
| *g__ML635J_40_aquatic_group* | CSBMs | 0.6121 | 9.04E-05 | Positive | |
| *g__UCG_005* | SAS | 0.6241 | 1.02E-04 | Positive | |
| *g__Klebsiella* | Straining | 0.6268 | 5.14E-05 | Positive | |
| *g__Parabacteroides* | Course of disease | 0.6270 | 5.14E-05 | Positive | |
| *g__Colidextribacter* | Course of disease | 0.6500 | 2.34E-05 | Positive | |
| *g__Eubacterium_ruminantium_group* | Course of disease | 0.6566 | 1.97E-05 | Positive | |
| *g__Intestinibacter* | SAS | 0.6594 | 3.14E-05 | Positive | |
| *g__Klebsiella* | Course of disease | 0.6647 | 1.42E-05 | Positive | |
| *g__UCG_002* | Course of disease | 0.6740 | 9.64E-06 | Positive | |
| *g__Intestinibacter* | SDS | 0.7019 | 8.33E-06 | Positive | |
| *g__Eubacterium_coprostanoligenes_group* | Course of disease | 0.7067 | 3.55E-06 | Positive | |
| *g__Akkermansia* | Course of disease | 0.7082 | 3.55E-06 | Positive | |
| *g__Intestinibacter* | Straining | 0.7199 | 3.25E-06 | Positive | |
| *g__Intestinibacter* | Course of disease | 0.7510 | 6.38E-07 | Positive | |

| **Table S3. The potential function of the gut microbiota.** | | | | |  |  |  |
| --- | --- | --- | --- | --- | --- | --- | --- |
| **Level 1** | **Level 2** | **avg(FC)** | **sd(FC)** | **avg(HC)** | **sd(HC)** | **p-value** | **FDR-value** |
| Genetic Information Processing | Replication and Repair | 0.086 | 0.002 | 0.087 | 0.003 | 0.026 | 0.124 |
| Metabolism | Energy Metabolism | 0.058 | 0.001 | 0.059 | 0.002 | 0.016 | 0.105 |
| Genetic Information Processing | Translation | 0.054 | 0.002 | 0.056 | 0.002 | 0.017 | 0.105 |
| Metabolism | Metabolism of Cofactors and Vitamins | 0.043 | 0.001 | 0.044 | 0.001 | 0.003 | 0.044 |
| Unclassified | Cellular Processes and Signaling | 0.042 | 0.001 | 0.041 | 0.001 | 0.003 | 0.044 |
| Metabolism | Nucleotide Metabolism | 0.038 | 0.001 | 0.039 | 0.002 | 0.021 | 0.114 |
| Metabolism | Lipid Metabolism | 0.029 | 0.001 | 0.027 | 0.001 | 0.001 | 0.023 |
| Unclassified | Metabolism | 0.024 | 0.001 | 0.023 | 0.001 | 0.015 | 0.105 |
| Organismal Systems | Excretory System | 0 | 0 | 0 | 0 | 0.004 | 0.044 |

| **Table S4. 42 up-regulated and 39 down-regulated differential metabolites were screened out.** | | | | |
| --- | --- | --- | --- | --- |
| **Metabolites** | **fold change** | ***p*-value** | **VIP-value** | **Regulation** |
| 4,7-dimethylpyrazolo[5,1-c][1,2,4]triazine-3-carbonitrile | 2.158 | 0.013 | 1.518 | Up |
| 4-Hydroxyhippuric acid | 2.879 | 0.002 | 1.800 | Up |
| 4-Methylphenol | 3.014 | 0.021 | 1.550 | Up |
| FAHFA (2:0/24:4) | 3.530 | 0.007 | 1.581 | Up |
| Glutaric acid | 5.602 | 0.000 | 2.438 | Up |
| Homoarginine | 2.007 | 0.001 | 1.984 | Up |
| L-Glutamine | 3.141 | 0.025 | 1.671 | Up |
| N-[4-(diethylamino)phenyl]-N'-phenylurea | 2.539 | 0.003 | 1.802 | Up |
| N2-Acetyl- L-lysine | 2.794 | 0.005 | 1.674 | Up |
| Prostaglandin E2 | 3.324 | 0.012 | 1.513 | Up |
| Prostaglandin K1 | 2.367 | 0.006 | 1.607 | Up |
| Norverapamil | 2.169 | 0.000 | 2.326 | Up |
| 2-(3,5-dimethyl-1H-pyrazol-4-yl)-5-methoxybenzoic acid | 2.018 | 0.000 | 2.096 | Up |
| N-cyclohexyl-2-(3-methyl-5-isoxazolyl)benzamide | 2.356 | 0.000 | 2.104 | Up |
| N-(2,3-dihydro-1,4-benzodioxin-6-yl)-2,5-dimethyl-3-furamide | 2.173 | 0.000 | 2.080 | Up |
| Pipecolic acid | 3.010 | 0.000 | 2.126 | Up |
| 2-(acetylamino)-3-(1H-indol-3-yl)propanoic acid | 2.085 | 0.000 | 1.900 | Up |
| N-(2,4-Dimethylphenyl)formamide | 2.346 | 0.001 | 1.920 | Up |
| N-Acetyl-L-tyrosine | 2.974 | 0.001 | 1.810 | Up |
| 3-Methyloxyindole | 2.038 | 0.001 | 1.770 | Up |
| 5-Hydroxytryptophol | 4.314 | 0.002 | 1.759 | Up |
| 1-(4-methyl-2-morpholino-1,3-thiazol-5-yl)ethan-1-one | 2.669 | 0.002 | 1.818 | Up |
| 2-[3-(4-pyridyl)-1H-1,2,4-triazol-5-yl]pyridine | 3.942 | 0.002 | 1.708 | Up |
| VMH | 2.641 | 0.003 | 1.685 | Up |
| Stercobilin | 2.452 | 0.003 | 1.694 | Up |
| 5-amino-2-(dimethylamino)benzoic acid | 2.163 | 0.003 | 1.730 | Up |
| Prostaglandin E2-1-glyceryl ester | 2.632 | 0.003 | 1.640 | Up |
| Lysopc 18:1 | 2.666 | 0.004 | 1.607 | Up |
| 1-[(1-benzylpiperidin-4-yl)amino]-3-[(4-fluorophenyl)thio]propan-2-ol | 2.715 | 0.004 | 1.653 | Up |
| Feruloyl Putrescine | 4.356 | 0.005 | 1.618 | Up |
| (3R)-4,4-Dimethyl-2-oxotetrahydro-3-furanyl beta-D-glucopyranoside | 2.496 | 0.005 | 1.562 | Up |
| 5-Methoxytryptamine | 2.608 | 0.005 | 1.560 | Up |
| Skatole | 8.049 | 0.006 | 1.575 | Up |
| Gatifloxacin | 2.850 | 0.006 | 1.568 | Up |
| ethyl 5-methoxy-2-methyl-1-phenyl-1H-indole-3-carboxylate | 3.029 | 0.006 | 1.534 | Up |
| methyl 2-[(2-acetyl-3-oxobut-1-enyl)amino]-3,4,5-trimethoxybenzoate | 3.297 | 0.007 | 1.538 | Up |
| N-(2-morpholinophenyl)-2-furamide | 2.105 | 0.007 | 1.543 | Up |
| Milbemycin A4 oxime | 2.379 | 0.009 | 1.587 | Up |
| N-phenyl-N'-{[5-(2-phenyleth-1-ynyl)-3-pyridyl]carbonyl}thiourea | 2.119 | 0.009 | 1.547 | Up |
| N-{5-[(dimethylamino)sulfonyl]-2-methylphenyl}cyclohexanecarboxamide | 3.021 | 0.012 | 1.523 | Up |
| Acetanilide | 2.076 | 0.014 | 1.557 | Up |
| 4-(5-propyl-2-pyridyl)benzonitrile | 2.044 | 0.033 | 1.607 | Up |
| 13,14-dihydro-19(R)-hydroxy Prostaglandin E1 | 0.302 | 0.005 | 1.660 | Down |
| Chenodeoxycholic Acid | 0.178 | 0.002 | 1.852 | Down |
| Glycocholic acid | 0.206 | 0.008 | 1.613 | Down |
| LPA 22:5 | 0.422 | 0.007 | 1.599 | Down |
| LPC 16:0 | 0.122 | 0.000 | 2.041 | Down |
| LPS 15:0 | 0.155 | 0.001 | 1.853 | Down |
| Soyasaponin I | 0.094 | 0.001 | 1.934 | Down |
| Stachyose | 0.280 | 0.010 | 1.585 | Down |
| (-)-Caryophyllene oxide | 0.106 | 0.000 | 2.146 | Down |
| Biliverdin | 0.314 | 0.000 | 1.972 | Down |
| 3-(4-pyridylmethylidene)chroman-4-one | 0.388 | 0.000 | 1.976 | Down |
| W-15-d4 | 0.405 | 0.000 | 1.969 | Down |
| Lysopc 18:3 | 0.103 | 0.001 | 1.913 | Down |
| 4-Pregnen-17alpha,20alpha-Diol-3-One | 0.434 | 0.001 | 1.827 | Down |
| Thromoboxane B1 | 0.172 | 0.001 | 1.761 | Down |
| LPE 18:2 | 0.167 | 0.002 | 1.781 | Down |
| Medroxyprogesterone | 0.126 | 0.002 | 1.722 | Down |
| Phytosphingosine | 0.133 | 0.002 | 1.762 | Down |
| Oleoyl ethanolamide | 0.273 | 0.003 | 1.687 | Down |
| 15-Deoxy-Δ12,14-prostaglandin A1 | 0.133 | 0.003 | 1.649 | Down |
| L-Argininosuccinate | 0.475 | 0.004 | 1.630 | Down |
| All-Trans-13,14-Dihydroretinol | 0.050 | 0.004 | 1.609 | Down |
| S-Adenosylmethionine | 0.209 | 0.006 | 1.579 | Down |
| 6-Deoxy-D-glucose | 0.448 | 0.006 | 1.580 | Down |
| α-Aspartylphenylalanine | 0.454 | 0.006 | 1.539 | Down |
| 3,3-dimethyl-2-morpholino-2,3-dihydrobenzo[b]furan-5-ol | 0.085 | 0.007 | 1.520 | Down |
| Methyl EudesMate | 0.388 | 0.007 | 1.610 | Down |
| gamma-Glutamylleucine | 0.455 | 0.007 | 1.532 | Down |
| S-Adenosyl-methionine | 0.214 | 0.007 | 1.540 | Down |
| Glycerophospho-N-palmitoyl ethanolamine | 0.305 | 0.007 | 1.772 | Down |
| 17beta-Trenbolone | 0.205 | 0.008 | 1.602 | Down |
| Guggulsterone | 0.404 | 0.010 | 1.539 | Down |
| Cytosine | 0.494 | 0.010 | 1.561 | Down |
| PC (14:1e/2:0) | 0.241 | 0.012 | 1.542 | Down |
| 3-Oxo-7alpha,12alpha-hydroxy-5beta-cholanoic acid | 0.116 | 0.012 | 1.537 | Down |
| Agmatine | 0.327 | 0.015 | 1.633 | Down |
| LPE 16:1 | 0.354 | 0.026 | 1.713 | Down |
| LPE 15:0 | 0.379 | 0.033 | 1.680 | Down |
| Epinephrine | 0.377 | 0.035 | 1.700 | Down |

| **Table S5. The correlations between differential metabolites and clinical parameters.** | | | | |
| --- | --- | --- | --- | --- |
| **Metabolites** | **Clinical parameters** | **r-value** | **FDR-value** | **Relation** |
| Feruloyl Putrescine | CSBMs | -0.730 | 1.64E-05 | Negative |
| Norverapamil | CSBMs | -0.711 | 2.59E-05 | Negative |
| N-(2,3-dihydro-1,4-benzodioxin-6-yl)-2,5-dimethyl-3-furamide | PAC-QOL | -0.695 | 1.10E-04 | Negative |
| Feruloyl Putrescine | PAC-QOL | -0.673 | 1.51E-04 | Negative |
| Glutaric acid | CSBMs | -0.668 | 1.10E-04 | Negative |
| Skatole | CSBMs | -0.667 | 1.10E-04 | Negative |
| N-(2,4-Dimethylphenyl)formamide | CSBMs | -0.657 | 1.51E-04 | Negative |
| N-cyclohexyl-2-(3-methyl-5-isoxazolyl)benzamide | CSBMs | -0.640 | 1.93E-04 | Negative |
| Norverapamil | BSFS | -0.638 | 1.93E-04 | Negative |
| 5-Methoxytryptamine | CSBMs | -0.624 | 2.73E-04 | Negative |
| 1-(4-methyl-2-morpholino-1,3-thiazol-5-yl)ethan-1-one | CSBMs | -0.617 | 3.10E-04 | Negative |
| N-{5-[(dimethylamino)sulfonyl]-2-methylphenyl}cyclohexanecarboxamide | CSBMs | -0.617 | 3.10E-04 | Negative |
| Stercobilin | CSBMs | -0.611 | 3.45E-04 | Negative |
| 5-Hydroxytryptophol | CSBMs | -0.611 | 3.45E-04 | Negative |
| 2-[3-(4-pyridyl)-1H-1,2,4-triazol-5-yl]pyridine | PAC-QOL | -0.605 | 5.25E-04 | Negative |
| 3-(4-pyridylmethylidene)chroman-4-one | Course of disease | -0.600 | 4.31E-04 | Negative |
| N-(2,4-Dimethylphenyl)formamide | PAC-QOL | -0.597 | 5.87E-04 | Negative |
| N-(2-morpholinophenyl)-2-furamide | PAC-QOL | -0.597 | 5.87E-04 | Negative |
| Guggulsterone | Course of disease | -0.596 | 4.57E-04 | Negative |
| Biliverdin | Course of disease | -0.594 | 4.85E-04 | Negative |
| 4-Hydroxyhippuric acid | PAC-QOL | -0.590 | 6.47E-04 | Negative |
| (3R)-4,4-Dimethyl-2-oxotetrahydro-3-furanyl beta-D-glucopyranoside | CSBMs | -0.587 | 5.25E-04 | Negative |
| Glycerophospho-N-palmitoyl ethanolamine | SAS | -0.585 | 7.50E-04 | Negative |
| 2-(3,5-dimethyl-1H-pyrazol-4-yl)-5-methoxybenzoic acid | PAC-QOL | -0.581 | 8.14E-04 | Negative |
| 2-(acetylamino)-3-(1H-indol-3-yl)propanoic acid | PAC-QOL | -0.577 | 8.86E-04 | Negative |
| Biliverdin | SDS | -0.576 | 8.86E-04 | Negative |
| Biliverdin | SAS | -0.575 | 8.87E-04 | Negative |
| Pipecolic acid | PAC-QOL | -0.572 | 9.26E-04 | Negative |
| Norverapamil | PAC-QOL | -0.569 | 9.96E-04 | Negative |
| N-Acetyl-L-tyrosine | PAC-QOL | -0.567 | 1.01E-03 | Negative |
| 3-(4-pyridylmethylidene)chroman-4-one | Straining | -0.562 | 8.14E-04 | Negative |
| 5-Methoxytryptamine | PAC-QOL | -0.559 | 1.19E-03 | Negative |
| ethyl 5-methoxy-2-methyl-1-phenyl-1H-indole-3-carboxylate | CSBMs | -0.558 | 8.86E-04 | Negative |
| 5-amino-2-(dimethylamino)benzoic acid | PAC-QOL | -0.557 | 1.21E-03 | Negative |
| Gatifloxacin | CSBMs | -0.555 | 9.07E-04 | Negative |
| 1-(4-methyl-2-morpholino-1,3-thiazol-5-yl)ethan-1-one | PAC-QOL | -0.553 | 1.32E-03 | Negative |
| 2-(acetylamino)-3-(1H-indol-3-yl)propanoic acid | CSBMs | -0.551 | 9.96E-04 | Negative |
| LPE 15:0 | SAS | -0.551 | 1.37E-03 | Negative |
| 3-Methyloxyindole | CSBMs | -0.550 | 9.96E-04 | Negative |
| LPE 16:1 | SAS | -0.550 | 1.37E-03 | Negative |
| 5-Hydroxytryptophol | PAC-QOL | -0.549 | 1.39E-03 | Negative |
| VMH | CSBMs | -0.548 | 1.03E-03 | Negative |
| Skatole | PAC-QOL | -0.546 | 1.44E-03 | Negative |
| Glycerophospho-N-palmitoyl ethanolamine | Course of disease | -0.542 | 1.19E-03 | Negative |
| S-Adenosylmethionine | Course of disease | -0.541 | 1.19E-03 | Negative |
| Phytosphingosine | Course of disease | -0.540 | 1.19E-03 | Negative |
| ethyl 5-methoxy-2-methyl-1-phenyl-1H-indole-3-carboxylate | PAC-QOL | -0.538 | 1.67E-03 | Negative |
| methyl 2-[(2-acetyl-3-oxobut-1-enyl)amino]-3,4,5-trimethoxybenzoate | PAC-QOL | -0.538 | 1.67E-03 | Negative |
| Milbemycin A4 oxime | CSBMs | -0.535 | 1.32E-03 | Negative |
| Biliverdin | Straining | -0.535 | 1.32E-03 | Negative |
| N2-Acetyl- L-lysine | PAC-QOL | -0.531 | 1.94E-03 | Negative |
| 3-Methyloxyindole | PAC-QOL | -0.525 | 2.13E-03 | Negative |
| Soyasaponin I | Straining | -0.523 | 1.59E-03 | Negative |
| (3R)-4,4-Dimethyl-2-oxotetrahydro-3-furanyl beta-D-glucopyranoside | PAC-QOL | -0.522 | 2.23E-03 | Negative |
| N-[4-(diethylamino)phenyl]-N'-phenylurea | CSBMs | -0.515 | 1.91E-03 | Negative |
| N-cyclohexyl-2-(3-methyl-5-isoxazolyl)benzamide | PAC-QOL | -0.514 | 2.61E-03 | Negative |
| Pipecolic acid | CSBMs | -0.514 | 1.94E-03 | Negative |
| N-(2-morpholinophenyl)-2-furamide | CSBMs | -0.512 | 2.00E-03 | Negative |
| (-)-Caryophyllene oxide | SAS | -0.511 | 2.71E-03 | Negative |
| Lysopc 18:1 | CSBMs | -0.510 | 2.05E-03 | Negative |
| N-[4-(diethylamino)phenyl]-N'-phenylurea | PAC-QOL | -0.510 | 2.76E-03 | Negative |
| 4-Hydroxyhippuric acid | CSBMs | -0.509 | 2.08E-03 | Negative |
| 2-[3-(4-pyridyl)-1H-1,2,4-triazol-5-yl]pyridine | CSBMs | -0.509 | 2.09E-03 | Negative |
| N-Acetyl-L-tyrosine | CSBMs | -0.509 | 2.09E-03 | Negative |
| Prostaglandin E2-1-glyceryl ester | CSBMs | -0.507 | 2.15E-03 | Negative |
| N-(2,3-dihydro-1,4-benzodioxin-6-yl)-2,5-dimethyl-3-furamide | BSFS | -0.505 | 2.22E-03 | Negative |
| Glutaric acid | PAC-QOL | -0.504 | 3.09E-03 | Negative |
| W-15-d4 | Course of disease | -0.501 | 2.36E-03 | Negative |
| Soyasaponin I | Course of disease | -0.500 | 2.41E-03 | Negative |
| LPC 16:0 | PAC-QOL | 0.502 | 3.17E-03 | Positive |
| 2-(acetylamino)-3-(1H-indol-3-yl)propanoic acid | Straining | 0.503 | 2.25E-03 | Positive |
| N-(2,3-dihydro-1,4-benzodioxin-6-yl)-2,5-dimethyl-3-furamide | Straining | 0.504 | 2.25E-03 | Positive |
| W-15-d4 | PAC-QOL | 0.504 | 3.09E-03 | Positive |
| 17beta-Trenbolone | CSBMs | 0.505 | 2.22E-03 | Positive |
| Oleoyl ethanolamide | CSBMs | 0.506 | 2.16E-03 | Positive |
| N-[4-(diethylamino)phenyl]-N'-phenylurea | Course of disease | 0.507 | 2.15E-03 | Positive |
| methyl 2-[(2-acetyl-3-oxobut-1-enyl)amino]-3,4,5-trimethoxybenzoate | SDS | 0.509 | 2.77E-03 | Positive |
| Medroxyprogesterone | CSBMs | 0.513 | 1.95E-03 | Positive |
| Prostaglandin E2 | Straining | 0.515 | 1.91E-03 | Positive |
| LPC 16:0 | CSBMs | 0.520 | 1.67E-03 | Positive |
| 4-Pregnen-17alpha,20alpha-Diol-3-One | CSBMs | 0.522 | 1.64E-03 | Positive |
| 3,3-dimethyl-2-morpholino-2,3-dihydrobenzo[b]furan-5-ol | CSBMs | 0.525 | 1.52E-03 | Positive |
| Cytosine | BSFS | 0.527 | 1.47E-03 | Positive |
| Glycerophospho-N-palmitoyl ethanolamine | CSBMs | 0.529 | 1.42E-03 | Positive |
| Lysopc 18:3 | PAC-QOL | 0.529 | 1.99E-03 | Positive |
| LPE 18:2 | CSBMs | 0.531 | 1.38E-03 | Positive |
| Glutaric acid | Straining | 0.532 | 1.38E-03 | Positive |
| Phytosphingosine | CSBMs | 0.533 | 1.37E-03 | Positive |
| 4-Hydroxyhippuric acid | Course of disease | 0.535 | 1.32E-03 | Positive |
| methyl 2-[(2-acetyl-3-oxobut-1-enyl)amino]-3,4,5-trimethoxybenzoate | SAS | 0.535 | 1.80E-03 | Positive |
| (3R)-4,4-Dimethyl-2-oxotetrahydro-3-furanyl beta-D-glucopyranoside | Course of disease | 0.537 | 1.28E-03 | Positive |
| Prostaglandin E2 | SAS | 0.543 | 1.52E-03 | Positive |
| 3-Oxo-7alpha,12alpha-hydroxy-5beta-cholanoic acid | CSBMs | 0.543 | 1.16E-03 | Positive |
| ethyl 5-methoxy-2-methyl-1-phenyl-1H-indole-3-carboxylate | SAS | 0.544 | 1.50E-03 | Positive |
| All-Trans-13,14-Dihydroretinol | CSBMs | 0.544 | 1.12E-03 | Positive |
| W-15-d4 | CSBMs | 0.546 | 1.07E-03 | Positive |
| Feruloyl Putrescine | Straining | 0.546 | 1.07E-03 | Positive |
| Soyasaponin I | PAC-QOL | 0.547 | 1.42E-03 | Positive |
| 3-Oxo-7alpha,12alpha-hydroxy-5beta-cholanoic acid | PAC-QOL | 0.547 | 1.42E-03 | Positive |
| 15-Deoxy-Δ12,14-prostaglandin A1 | CSBMs | 0.556 | 8.97E-04 | Positive |
| 2-[3-(4-pyridyl)-1H-1,2,4-triazol-5-yl]pyridine | Course of disease | 0.557 | 8.87E-04 | Positive |
| Norverapamil | Course of disease | 0.563 | 8.14E-04 | Positive |
| 3-Methyloxyindole | Course of disease | 0.564 | 7.96E-04 | Positive |
| ethyl 5-methoxy-2-methyl-1-phenyl-1H-indole-3-carboxylate | Course of disease | 0.569 | 6.94E-04 | Positive |
| 5-Hydroxytryptophol | Course of disease | 0.572 | 6.47E-04 | Positive |
| (-)-Caryophyllene oxide | CSBMs | 0.574 | 6.31E-04 | Positive |
| 2-(acetylamino)-3-(1H-indol-3-yl)propanoic acid | Course of disease | 0.574 | 6.31E-04 | Positive |
| N-(2-morpholinophenyl)-2-furamide | Course of disease | 0.574 | 6.31E-04 | Positive |
| N-cyclohexyl-2-(3-methyl-5-isoxazolyl)benzamide | Course of disease | 0.574 | 6.31E-04 | Positive |
| 3-(4-pyridylmethylidene)chroman-4-one | BSFS | 0.578 | 5.87E-04 | Positive |
| 5-Methoxytryptamine | Course of disease | 0.581 | 5.76E-04 | Positive |
| N-(2,4-Dimethylphenyl)formamide | Course of disease | 0.582 | 5.62E-04 | Positive |
| Lysopc 18:3 | CSBMs | 0.585 | 5.31E-04 | Positive |
| N-(2,3-dihydro-1,4-benzodioxin-6-yl)-2,5-dimethyl-3-furamide | Course of disease | 0.590 | 5.13E-04 | Positive |
| Thromoboxane B1 | CSBMs | 0.590 | 5.13E-04 | Positive |
| N-Acetyl-L-tyrosine | Course of disease | 0.592 | 4.95E-04 | Positive |
| Skatole | Course of disease | 0.596 | 4.57E-04 | Positive |
| Pipecolic acid | Course of disease | 0.601 | 4.23E-04 | Positive |
| Biliverdin | PAC-QOL | 0.602 | 5.62E-04 | Positive |
| Biliverdin | CSBMs | 0.602 | 4.23E-04 | Positive |
| 3-(4-pyridylmethylidene)chroman-4-one | PAC-QOL | 0.606 | 5.25E-04 | Positive |
| Norverapamil | Straining | 0.608 | 3.75E-04 | Positive |
| Chenodeoxycholic Acid | CSBMs | 0.616 | 3.10E-04 | Positive |
| Guggulsterone | PAC-QOL | 0.622 | 4.23E-04 | Positive |
| Guggulsterone | CSBMs | 0.633 | 1.99E-04 | Positive |
| Feruloyl Putrescine | Course of disease | 0.635 | 1.93E-04 | Positive |
| 3-(4-pyridylmethylidene)chroman-4-one | CSBMs | 0.636 | 1.93E-04 | Positive |
| 1-(4-methyl-2-morpholino-1,3-thiazol-5-yl)ethan-1-one | Course of disease | 0.638 | 1.93E-04 | Positive |
| Soyasaponin I | CSBMs | 0.640 | 1.93E-04 | Positive |

| **Table S6. The main correlations of differential genera and differential metabolites (r > 0.5, FDR < 0.05).** | | | | |
| --- | --- | --- | --- | --- |
| **Genus** | **Metabolites** | **r-value** | **FDR-value** | **Relation** |
| *g__UCG_005* | (-)-Caryophyllene oxide | -0.784 | 8.17E-07 | Negative |
| *g__Eubacterium_ruminantium_group* | Glycerophospho-N-palmitoyl ethanolamine | -0.716 | 3.57E-05 | Negative |
| *g__UCG_005* | Glycerophospho-N-palmitoyl ethanolamine | -0.715 | 3.57E-05 | Negative |
| *g__UCG_005* | Lysopc 18:3 | -0.706 | 4.47E-05 | Negative |
| *g__Eubacterium_coprostanoligenes_group* | (-)-Caryophyllene oxide | -0.666 | 1.35E-04 | Negative |
| *g__Eubacterium_coprostanoligenes_group* | Glycerophospho-N-palmitoyl ethanolamine | -0.646 | 2.27E-04 | Negative |
| *g__Akkermansia* | Glycerophospho-N-palmitoyl ethanolamine | -0.645 | 2.27E-04 | Negative |
| *g__Lachnoclostridium* | Guggulsterone | -0.641 | 2.64E-04 | Negative |
| *g__Eubacterium_ruminantium_group* | (-)-Caryophyllene oxide | -0.636 | 3.02E-04 | Negative |
| *g__Eubacterium_coprostanoligenes_group* | Lysopc 18:3 | -0.630 | 3.45E-04 | Negative |
| *g__Incertae_Sedis* | LPE 18:2 | -0.627 | 3.75E-04 | Negative |
| *g__UCG_002* | Thromoboxane B1 | -0.626 | 3.76E-04 | Negative |
| *g__Intestinibacter* | Biliverdin | -0.625 | 3.76E-04 | Negative |
| *g__Eubacterium_coprostanoligenes_group* | Thromoboxane B1 | -0.620 | 4.00E-04 | Negative |
| *g__UCG_002* | Glycerophospho-N-palmitoyl ethanolamine | -0.619 | 4.00E-04 | Negative |
| *g__Colidextribacter* | LPE 18:2 | -0.617 | 4.00E-04 | Negative |
| *g__UCG_002* | 15-Deoxy-Δ12,14-prostaglandin A1 | -0.616 | 4.05E-04 | Negative |
| *g__UCG_002* | All-Trans-13,14-Dihydroretinol | -0.612 | 4.28E-04 | Negative |
| *g__UCG_002* | (-)-Caryophyllene oxide | -0.608 | 4.96E-04 | Negative |
| *g__UCG_005* | LPE 18:2 | -0.606 | 5.16E-04 | Negative |
| *g__Eubacterium_ruminantium_group* | N-(2,4-Dimethylphenyl)formamide | 0.605 | 5.20E-04 | Positive |
| *g__Eubacterium_ruminantium_group* | N-Acetyl-L-tyrosine | 0.612 | 4.28E-04 | Positive |
| *g__Incertae_Sedis* | N-[4-(diethylamino)phenyl]-N'-phenylurea | 0.612 | 4.28E-04 | Positive |
| *g__Eubacterium_coprostanoligenes_group* | N-[4-(diethylamino)phenyl]-N'-phenylurea | 0.614 | 4.21E-04 | Positive |
| *g__UCG_005* | N-Acetyl-L-tyrosine | 0.617 | 4.00E-04 | Positive |
| *g__Eubacterium_coprostanoligenes_group* | N-(2,4-Dimethylphenyl)formamide | 0.618 | 4.00E-04 | Positive |
| *g__UCG_005* | ethyl 5-methoxy-2-methyl-1-phenyl-1H-indole-3-carboxylate | 0.619 | 4.00E-04 | Positive |
| *g__Lachnospiraceae_NK4A136_group* | N-[4-(diethylamino)phenyl]-N'-phenylurea | 0.621 | 4.00E-04 | Positive |
| *g__Eubacterium_coprostanoligenes_group* | Skatole | 0.623 | 3.86E-04 | Positive |
| *g__UCG_005* | Skatole | 0.623 | 3.86E-04 | Positive |
| *g__Akkermansia* | N-Acetyl-L-tyrosine | 0.633 | 3.20E-04 | Positive |
| *g__Eubacterium_ruminantium_group* | 1-(4-methyl-2-morpholino-1,3-thiazol-5-yl)ethan-1-one | 0.633 | 3.20E-04 | Positive |
| *g__UCG_002* | Skatole | 0.646 | 2.27E-04 | Positive |
| *g__UCG_005* | 1-(4-methyl-2-morpholino-1,3-thiazol-5-yl)ethan-1-one | 0.648 | 2.27E-04 | Positive |
| *g__Eubacterium_coprostanoligenes_group* | N-cyclohexyl-2-(3-methyl-5-isoxazolyl)benzamide | 0.650 | 2.27E-04 | Positive |
| *g__UCG_002* | 1-(4-methyl-2-morpholino-1,3-thiazol-5-yl)ethan-1-one | 0.659 | 1.66E-04 | Positive |
| *g__Intestinibacter* | Norverapamil | 0.665 | 1.35E-04 | Positive |
| *g__Eubacterium_coprostanoligenes_group* | N-Acetyl-L-tyrosine | 0.668 | 1.35E-04 | Positive |
| *g__Eubacterium_coprostanoligenes_group* | 1-(4-methyl-2-morpholino-1,3-thiazol-5-yl)ethan-1-one | 0.669 | 1.35E-04 | Positive |
| *g__UCG_002* | N-cyclohexyl-2-(3-methyl-5-isoxazolyl)benzamide | 0.678 | 1.04E-04 | Positive |
| *g__Colidextribacter* | N-[4-(diethylamino)phenyl]-N'-phenylurea | 0.679 | 1.04E-04 | Positive |
| *g__UCG_005* | Gatifloxacin | 0.679 | 1.04E-04 | Positive |
| *g__Eubacterium_coprostanoligenes_group* | Feruloyl Putrescine | 0.696 | 6.22E-05 | Positive |

| **Table S7. AUC-values of the 18 differential genera.** | | | |
| --- | --- | --- | --- |
| **Genus** | **AUC-value** | **95% Confidence Interval** | ***p*-value** |
| *g__Intestinibacter* | 0.9728 | 91.89%~ 100% | <0.0001 |
| *g__Eubacterium_coprostanoligenes_group* | 0.9501 | 88.79% ~ 100% | <0.0001 |
| *g__Klebsiella* | 0.9263 | 84.52% ~ 100% | <0.0001 |
| *g__Colidextribacter* | 0.9116 | 82.64% ~ 99.68% | <0.0001 |
| *g__Eubacterium_ruminantium_group* | 0.9002 | 80.05% ~ 100% | <0.0001 |
| *g__Akkermansia* | 0.8957 | 78.60% ~ 100% | <0.0001 |
| *g__UCG_002* | 0.8912 | 79.56% ~ 98.67% | <0.0001 |
| *g__Incertae_Sedis* | 0.8685 | 75.29% ~ 98.41% | <0.0001 |
| *g__UCG_005* | 0.8662 | 75.57% ~ 97.67% | <0.0001 |
| *g__Parabacteroides* | 0.8367 | 70.62% ~ 96.73% | 0.0002 |
| *g__Lachnoclostridium* | 0.8356 | 70.12% ~ 97.00% | 0.0002 |
| *g__ML635J_40_aquatic_group* | 0.8095 | 67.08% ~ 94.83% | 0.0006 |
| *g__Lachnospiraceae_NK4A136_group* | 0.7925 | 65.87% ~ 92.63% | 0.0012 |
| *g__Lachnospira* | 0.7868 | 64.60% ~ 92.77% | 0.0015 |
| *g__Eubacterium_hallii_group* | 0.7732 | 62.83% ~ 91.82% | 0.0024 |
| *g__Barnesiella* | 0.7540 | 60.66% ~ 90.14% | 0.0048 |
| *g__Eubacterium_eligens_group* | 0.7256 | 56.58% ~ 88.54% | 0.0123 |
| *g__Gilliamella* | 0.6190 | 44.75% ~ 79.06% | 0.1866 |

| **Table S8. AUC-values of the 57 differential metabolites.** | | | |
| --- | --- | --- | --- |
| **Metabolites** | **AUC-value** | **95% Confidence Interval** | ***p*-value** |
| Feruloyl Putrescine | 0.8753 | 76.42% ~ 98.64% | <0.0001 |
| Norverapamil | 0.8685 | 75.45% ~ 98.25% | <0.0001 |
| 3-(4-pyridylmethylidene)chroman-4-one | 0.8662 | 75.13% ~ 98.11% | <0.0001 |
| Biliverdin | 0.8639 | 75.70% ~ 97.09% | <0.0001 |
| Glutaric acid | 0.8594 | 74.03% ~ 97.85% | <0.0001 |
| Skatole | 0.8526 | 73.90% ~ 96.62% | <0.0001 |
| 5-Methoxytryptamine | 0.8503 | 72.98% ~ 97.09% | 0.0001 |
| Soyasaponin I | 0.8481 | 72.40% ~ 97.21% | 0.0001 |
| Pipecolic acid | 0.8458 | 71.98% ~ 97.18% | 0.0001 |
| Guggulsterone | 0.8458 | 72.13% ~ 97.03% | 0.0001 |
| N-(2,4-Dimethylphenyl)formamide | 0.8458 | 72.62% ~ 96.54% | 0.0001 |
| N-(2,3-dihydro-1,4-benzodioxin-6-yl)-2,5-dimethyl-3-furamide | 0.8458 | 72.59% ~ 96.57% | 0.0001 |
| ethyl 5-methoxy-2-methyl-1-phenyl-1H-indole-3-carboxylate | 0.8435 | 72.43% ~ 96.28% | 0.0001 |
| N-cyclohexyl-2-(3-methyl-5-isoxazolyl)benzamide | 0.8435 | 72.12% ~ 96.59% | 0.0001 |
| N-(2-morpholinophenyl)-2-furamide | 0.8345 | 70.75% ~ 96.14% | 0.0002 |
| 5-Hydroxytryptophol | 0.8345 | 71.45% ~ 95.44% | 0.0002 |
| N-Acetyl-L-tyrosine | 0.8345 | 71.13% ~ 95.76% | 0.0002 |
| 1-(4-methyl-2-morpholino-1,3-thiazol-5-yl)ethan-1-one | 0.8345 | 70.72% ~ 96.17% | 0.0002 |
| (3R)-4,4-Dimethyl-2-oxotetrahydro-3-furanyl beta-D-glucopyranoside | 0.8345 | 71.25% ~ 95.64% | 0.0002 |
| 2-(acetylamino)-3-(1H-indol-3-yl)propanoic acid | 0.8322 | 71.04% ~ 95.40% | 0.0002 |
| 2-(3,5-dimethyl-1H-pyrazol-4-yl)-5-methoxybenzoic acid | 0.8299 | 70.70% ~ 95.29% | 0.0003 |
| Lysopc 18:3 | 0.8277 | 69.68% ~ 95.85% | 0.0003 |
| (-)-Caryophyllene oxide | 0.8209 | 68.50% ~ 95.68% | 0.0004 |
| Phytosphingosine | 0.8209 | 69.62% ~ 94.55% | 0.0004 |
| S-Adenosylmethionine | 0.8209 | 69.49% ~ 94.68% | 0.0004 |
| N-[4-(diethylamino)phenyl]-N'-phenylurea | 0.8209 | 69.00% ~ 95.17% | 0.0004 |
| 3-Methyloxyindole | 0.8163 | 69.09% ~ 94.18% | 0.0004 |
| 4-Hydroxyhippuric acid | 0.8163 | 68.94% ~ 94.33% | 0.0004 |
| Gatifloxacin | 0.8141 | 68.40% ~ 94.42% | 0.0005 |
| 5-amino-2-(dimethylamino)benzoic acid | 0.8095 | 67.64% ~ 94.26% | 0.0006 |
| W-15-d4 | 0.8073 | 67.00% ~ 94.45% | 0.0007 |
| Thromoboxane B1 | 0.8027 | 66.94% ~ 93.60% | 0.0008 |
| 2-[3-(4-pyridyl)-1H-1,2,4-triazol-5-yl]pyridine | 0.8027 | 66.90% ~ 93.64% | 0.0008 |
| Glycerophospho-N-palmitoyl ethanolamine | 0.8005 | 66.15% ~ 93.94% | 0.0009 |
| Stercobilin | 0.7959 | 65.67% ~ 93.51% | 0.001 |
| methyl 2-[(2-acetyl-3-oxobut-1-enyl)amino]-3,4,5-trimethoxybenzoate | 0.7937 | 65.57% ~ 93.16% | 0.0011 |
| Chenodeoxycholic Acid | 0.7937 | 65.49% ~ 93.24% | 0.0011 |
| N2-Acetyl- L-lysine | 0.7914 | 65.20% ~ 93.07% | 0.0012 |
| Prostaglandin E2-1-glyceryl ester | 0.7891 | 64.97% ~ 92.85% | 0.0013 |
| Medroxyprogesterone | 0.7868 | 63.87% ~ 93.50% | 0.0015 |
| LPE 18:2 | 0.7846 | 64.27% ~ 92.65% | 0.0016 |
| LPC 16:0 | 0.7846 | 63.49% ~ 93.42% | 0.0016 |
| Lysopc 18:1 | 0.7823 | 63.89% ~ 92.57% | 0.0017 |
| All-Trans-13,14-Dihydroretinol | 0.7800 | 62.98% ~ 93.03% | 0.0019 |
| 3,3-dimethyl-2-morpholino-2,3-dihydrobenzo[b]furan-5-ol | 0.7732 | 62.99% ~ 91.66% | 0.0024 |
| 15-Deoxy-Δ12,14-prostaglandin A1 | 0.7710 | 62.65% ~ 91.55% | 0.0026 |
| S-Adenosyl-methionine | 0.7710 | 62.59% ~ 91.60% | 0.0026 |
| Prostaglandin E2 | 0.7710 | 62.66% ~ 91.54% | 0.0026 |
| LPS 15:0 | 0.7710 | 62.80% ~ 91.39% | 0.0026 |
| 1-[(1-benzylpiperidin-4-yl)amino]-3-[(4-fluorophenyl)thio]propan-2-ol | 0.7687 | 62.14% ~ 91.60% | 0.0029 |
| L-Argininosuccinate | 0.7687 | 62.50% ~ 91.24% | 0.0029 |
| Acetanilide | 0.7664 | 61.57% ~ 91.72% | 0.0031 |
| VMH | 0.7642 | 61.51% ~ 91.32% | 0.0034 |
| PC (14:1e/2:0) | 0.7619 | 61.95% ~ 90.43% | 0.0037 |
| 3-Oxo-7alpha,12alpha-hydroxy-5beta-cholanoic acid | 0.7619 | 60.94% ~ 91.44% | 0.0037 |
| Cytosine | 0.7596 | 61.24% ~ 90.69% | 0.004 |
| N-{5-[(dimethylamino)sulfonyl]-2-methylphenyl}cyclohexanecarboxamide | 0.7574 | 60.87% ~ 90.60% | 0.0043 |
| Homoarginine | 0.7574 | 59.89% ~ 91.58% | 0.0043 |
| N-phenyl-N'-{[5-(2-phenyleth-1-ynyl)-3-pyridyl]carbonyl}thiourea | 0.7551 | 60.82% ~ 90.20% | 0.0047 |
| 17beta-Trenbolone | 0.7506 | 59.45% ~ 90.66% | 0.0054 |
| Stachyose | 0.7506 | 60.18% ~ 89.93% | 0.0054 |
| Agmatine | 0.7483 | 60.15% ~ 89.51% | 0.0059 |
| 4-Pregnen-17alpha,20alpha-Diol-3-One | 0.7483 | 59.36% ~ 90.30% | 0.0059 |
| Milbemycin A4 oxime | 0.7415 | 58.28% ~ 90.02% | 0.0074 |
| 4,7-dimethylpyrazolo[5,1-c][1,2,4]triazine-3-carbonitrile | 0.7415 | 58.80% ~ 89.50% | 0.0074 |
| Prostaglandin K1 | 0.7415 | 59.21% ~ 89.09% | 0.0074 |
| Oleoyl ethanolamide | 0.7347 | 57.65% ~ 89.29% | 0.0092 |
| gamma-Glutamylleucine | 0.7302 | 57.41% ~ 88.62% | 0.0107 |
| 6-Deoxy-D-glucose | 0.7302 | 57.40% ~ 88.63% | 0.0107 |
| LPA 22:5 | 0.7279 | 56.80% ~ 88.77% | 0.0115 |
| LPE 15:0 | 0.7188 | 55.84% ~ 87.92% | 0.0152 |
| α-Aspartylphenylalanine | 0.7166 | 56.16% ~ 87.15% | 0.0163 |
| 13,14-dihydro-19(R)-hydroxy Prostaglandin E1 | 0.7120 | 55.27% ~ 87.13% | 0.0187 |
| FAHFA (2:0/24:4) | 0.7098 | 55.44% ~ 86.51% | 0.02 |
| 4-(5-propyl-2-pyridyl)benzonitrile | 0.6961 | 53.51% ~ 85.71% | 0.0296 |
| Glycocholic acid | 0.6893 | 51.53% ~ 86.34% | 0.0357 |
| LPE 16:1 | 0.6825 | 52.19% ~ 84.32% | 0.0429 |
| Methyl EudesMate | 0.6780 | 51.27% ~ 84.33% | 0.0483 |
| 4-Methylphenol | 0.6780 | 51.19% ~ 84.41% | 0.0483 |
| L-Glutamine | 0.5805 | 40.62% ~ 75.48% | 0.3718 |
| Epinephrine | 0.5420 | 35.30% ~ 73.09% | 0.6417 |
